# A Bibliometric and In Silico-Based Analysis of Anti-Lung Cancer Compounds from Sea Cucumber

**DOI:** 10.3390/md21050283

**Published:** 2023-04-28

**Authors:** Afshin Zare, Safoura Izanloo, Sajed Khaledi, Mussin Nadiar Maratovich, Asset Askerovich Kaliyev, Nurgul Abdullayevna Abenova, Farhad Rahmanifar, Mahdi Mahdipour, Shabnam Bakhshalizadeh, Reza Shirazi, Nader Tanideh, Amin Tamadon

**Affiliations:** 1 The PerciaVista Biotechnology Company, Shiraz 71676-83745, Iran; afshinzareresearch@gmail.com (A.Z.);; 2 Department of Anatomical Sciences, School of Medicine, Iran University of Medical Sciences, Tehran 14496-14535, Iran; 3 School of Nursing, North Khorasan University of Medical Sciences, Bojnurd 94149-74877, Iran; 4 Department of Anatomy, Faculty of Medical Sciences, Tarbiat Modares University, Tehran 14117-13116, Iran; 5 General Surgery, West Kazakhstan Marat Ospanov Medical University, Aktobe 030019, Kazakhstan; nadiar_musin@zkmu.kz (M.N.M.); aset_kaliyev@mail.ru (A.A.K.); 6 Department of Internal Diseases, West Kazakhstan Marat Ospanov Medical University, Aktobe 030019, Kazakhstan; 7 Department of Basic Sciences, School of Veterinary Medicine, Shiraz University, Shiraz 71348-14336, Iran; 8 Stem Cell Research Center, Tabriz University of Medical Sciences, Tabriz 51666-53431, Iran; 9 Department of Applied Cell Sciences, Faculty of Advanced Medical Sciences, Tabriz University of Medical Sciences, Tabriz 51666-53431, Iran; 10 Reproductive Development, Murdoch Children’s Research Institute, Melbourne, VIC 3052, Australia; 11 Department of Paediatrics, University of Melbourne, Melbourne, VIC 3010, Australia; 12 Department of Anatomy, School of Medical Sciences, Biomedical & Health, UNSW Sydney, Sydney, NSW 1466, Australia; reza.shirazi@unsw.edu.au; 13 Stem Cells Technology Research Center, Shiraz University of Medical Sciences, Shiraz 71348-14336, Iran; 14 Department of Pharmacology, Medical School, Shiraz University of Medical Sciences, Shiraz 71348-14336, Iran; 15 Department for Scientific Work, West Kazakhstan Marat Ospanov Medical University, Aktobe 030010, Kazakhstan

**Keywords:** lung neoplasms, marine biology, bibliometrics, in silico techniques, sea cucumbers, anti-cancer agents, triterpenes, glucosides, apoptosis

## Abstract

Lung cancer is one of the most lethal malignancies in the world. However, current curative approaches for treating this type of cancer have some weaknesses. Therefore, scientists are attempting to discover new anti-lung cancer agents. Sea cucumber is a marine-derived source for discovering biologically active compounds with anti-lung cancer properties. To explore the anti-lung cancer properties of sea cucumber, we analyzed surveys using VOSviewer software and identified the most frequently used keywords. We then searched the Google Scholar database for compounds with anti-lung cancer properties within that keyword family. Finally, we used AutoDock 4 to identify the compounds with the highest affinity for apoptotic receptors in lung cancer cells. The results showed that triterpene glucosides were the most frequently identified compounds in studies examining the anti-cancer properties of sea cucumbers. Intercedenside C, Scabraside A, and Scabraside B were the three triterpene glycosides with the highest affinity for apoptotic receptors in lung cancer cells. To the best of our knowledge, this is the first time that anti-lung cancer properties of sea cucumber-derived compounds have been examined in in silico conditions. Ultimately, these three components displayed anti-lung cancer properties in in silico conditions and may be used for the manufacture of anti-lung cancer agents in the near future.

## 1. Introduction

To date, lung cancer is known as the most commonly diagnosed cancer with a high mortality rate, making it vital to develop effective anti-tumor agents [1]. Although surgery, chemotherapy, radiotherapy, and adjuvant therapy are the most utilized methods to treat this malady [2], some of their weak points include resistance against current medications, toxicity of current therapeutic agents, lack of publicity for different types of treatment strategies, and the lack of a certain curative drug, all of which have urged scientists to search for novel compounds with anti-lung cancer properties [2].

Natural sources have gained a lot of attention from scientists in order to find new biologically active components [3]. The marine environment is a rich source of discovery for novel therapeutic anti-cancer agents due to the vast diversity of its biological components. As a result, approximately 14,000 pharmacologically active components have been discovered and isolated from them. Therefore, scientists are attracted to marine-derived sources with the purpose of treating malignancies [4,5,6].

Sea cucumbers are invertebrates known to be valuable marine sources with a vast range of uses in the food and medical industries. Previous studies have demonstrated that sea cucumbers have various biologically active compounds, including proteins, cerebrosides, triterpene glycosides, sphingoids, and polysaccharides [7]. Their extract has exhibited anti-inflammatory, anti-oxidant, and anti-tumor activities [8].

Various types of in vivo, in vitro, and in silico studies have been conducted to discover the accurate anti-tumor mechanisms of sea cucumbers. The results have shown that sea cucumbers and their extract can induce cancer cell death via apoptosis induction, inhibiting cell division and inducing anti-angiogenesis effects [3].

Due to the insufficient amount of review studies in the field of anti-lung cancer features of compounds in sea cucumbers, this study examines all the research in the field of anti-lung cancer effects of sea cucumbers to find the most frequent biologically active compounds in the extracts of different types of sea cucumbers with anti-lung cancer properties. After that, we assessed the affinity of these components to the receptors involved in the death of lung cancer cells using molecular docking. Finally, this study identifies the most potent anti-lung cancer compound in the extracts of sea cucumbers based on previous surveys and an in silico condition. To the best of our knowledge, this manuscript is the first study that examines anti-lung cancer compounds of sea cucumbers in an in silico condition. These compounds may be used to develop novel anti-lung cancer drugs in the near future.

## 2. Results

### 2.1. PubMed Online Database Analysis

**Figure 1:**
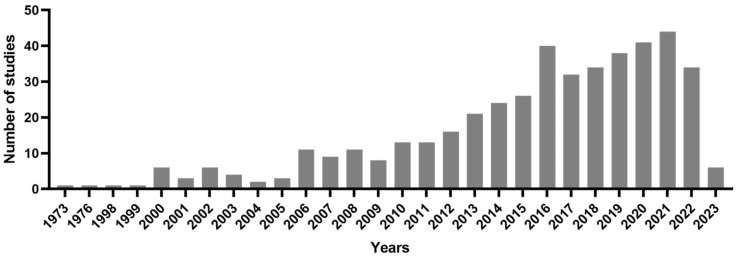
The number of studies examining the anti-cancer effects of sea cucumber from 1973 to 2023.

An analysis of the PubMed online database revealed 398 surveys conducted between 1973 and 2023 that have examined the anti-cancer properties of sea cucumbers. The number of studies on sea cucumber’s anti-cancer properties is depicted in Figure 1.

### 2.2. Triterpene Glycosides Are the Most Frequent Components in Anti-Cancer Studies of Sea Cucumber

**Figure 2:**
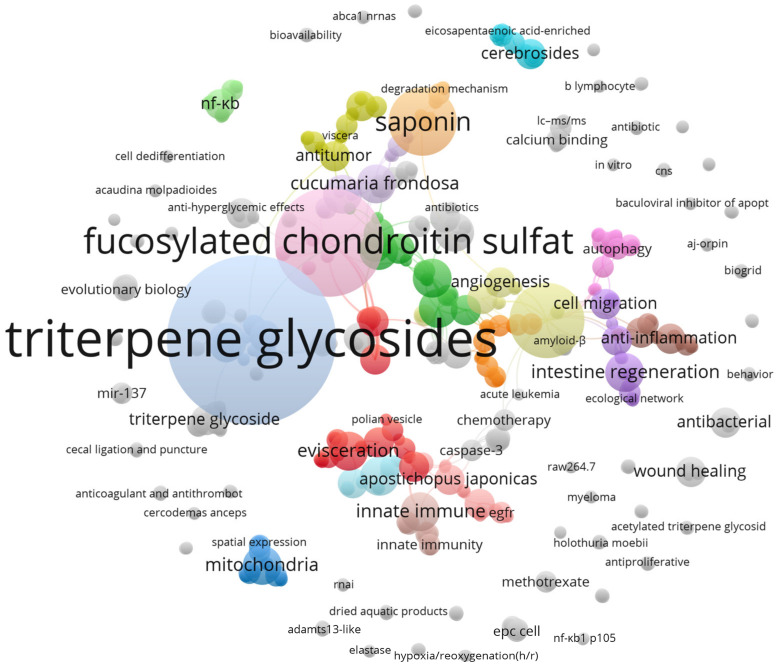
The most frequent keywords in the surveys that have studied the anti-cancer features of sea cucumber. The size of each keyword represents the frequency of it.

VOSviewer software analysis categorized all 398 studies into 703 items, 84 clusters, 1694 links, with a total link strength of 1768. Moreover, according to the analysis of the VOSviewer software on all 398 surveys that had investigated the anticancer features of sea cucumbers, it was found that triterpene glycosides were the most frequently occurring type of compound in various types of sea cucumbers that have been studied for their anti-cancer features (Figure 2 and Table 1). After triterpene glycosides, Fucosylated chondroitin sulfate and Frondoside A ranked second and third, respectively (Table 1).

### 2.3. Triterpene Glycosides in the Sea Cucumber Extract with Anti-Lung Cancer Properties

Since 1973, 22 triterpene glycosides have been proven to possess anti-lung cancer properties. Detailed information regarding the effects of all 22 mentioned components on lung cancer can be found in Table 2.

### 2.4. Intercedenside C, Scabraside A, and Scabraside B Had the Most Affinity to Receptors That Are Involved in the Apoptosis of Lung Cancer Cells

**Figure 3:**
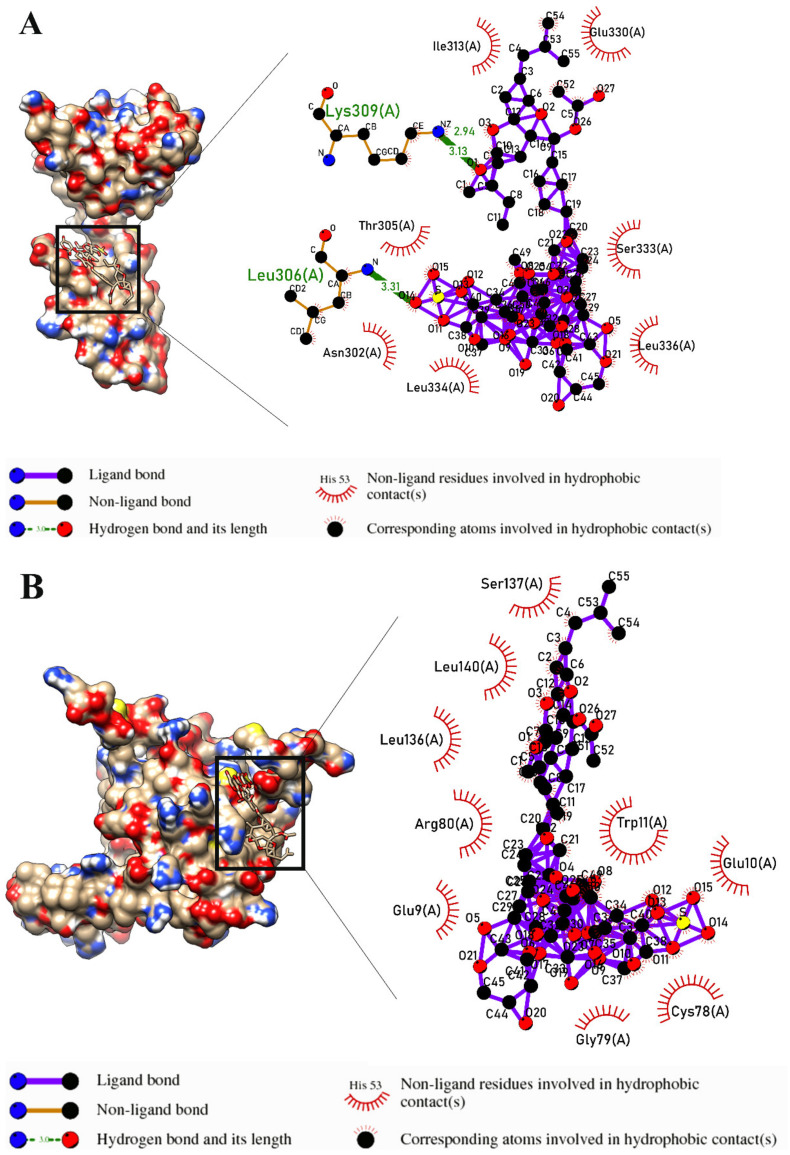

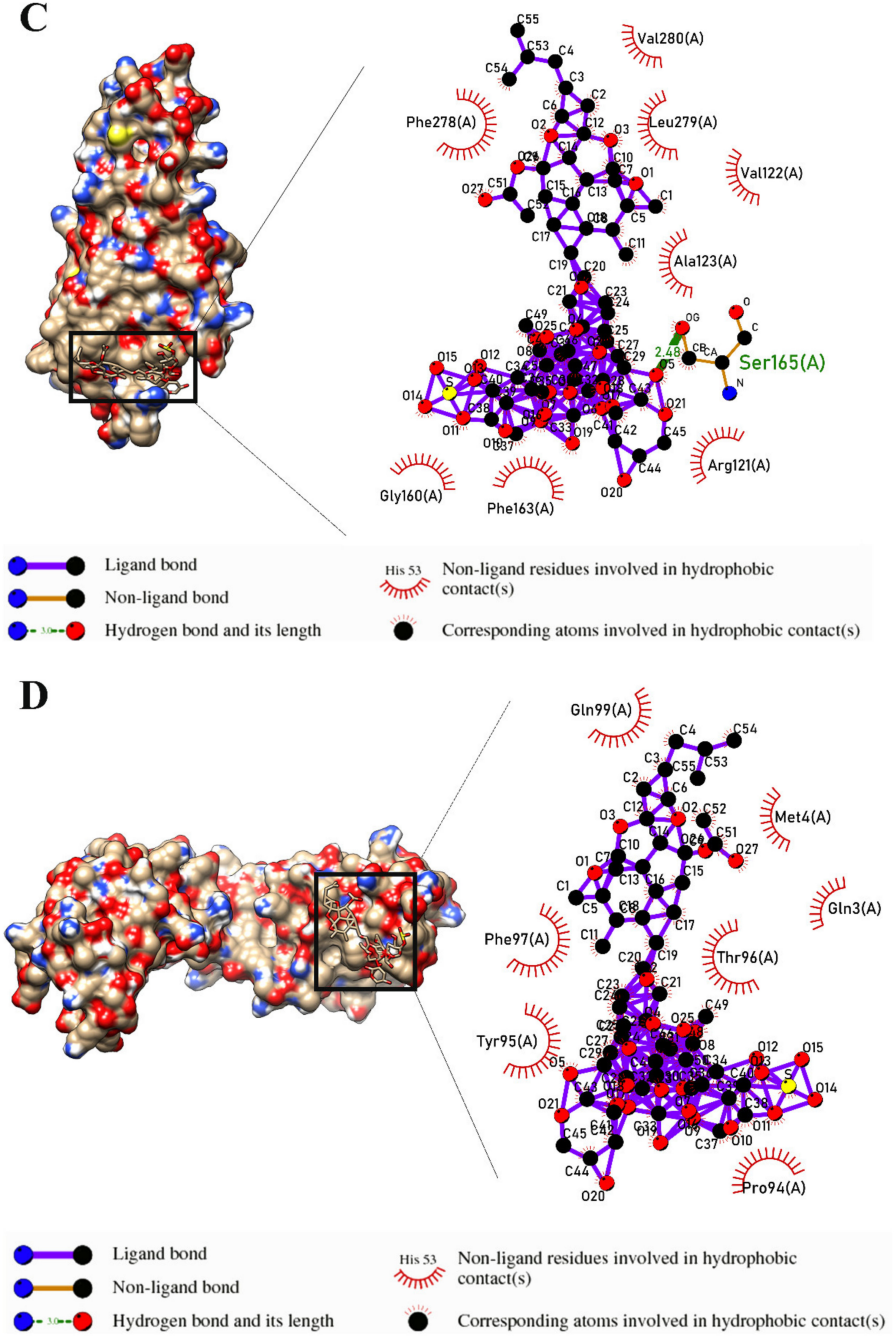

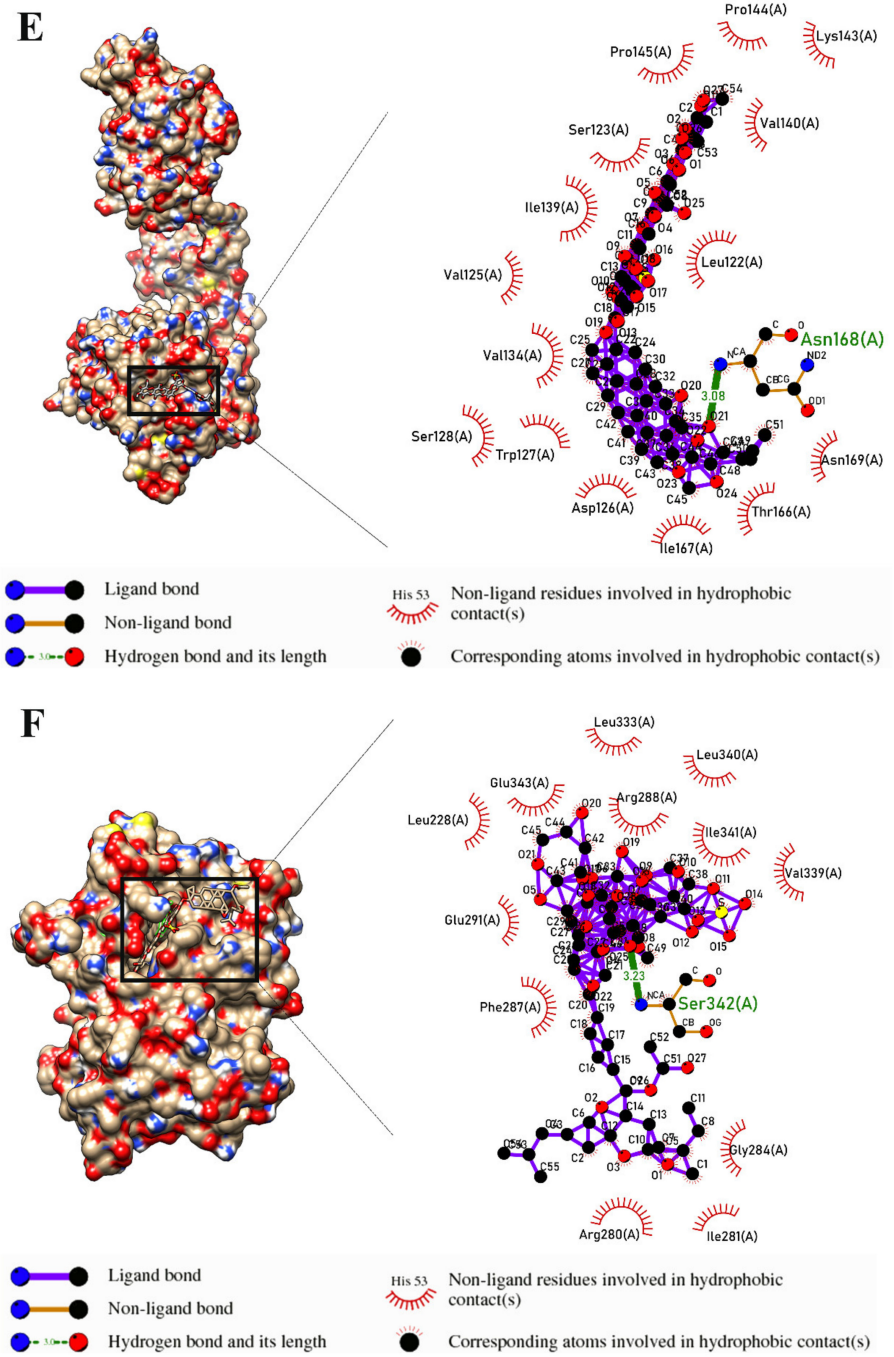

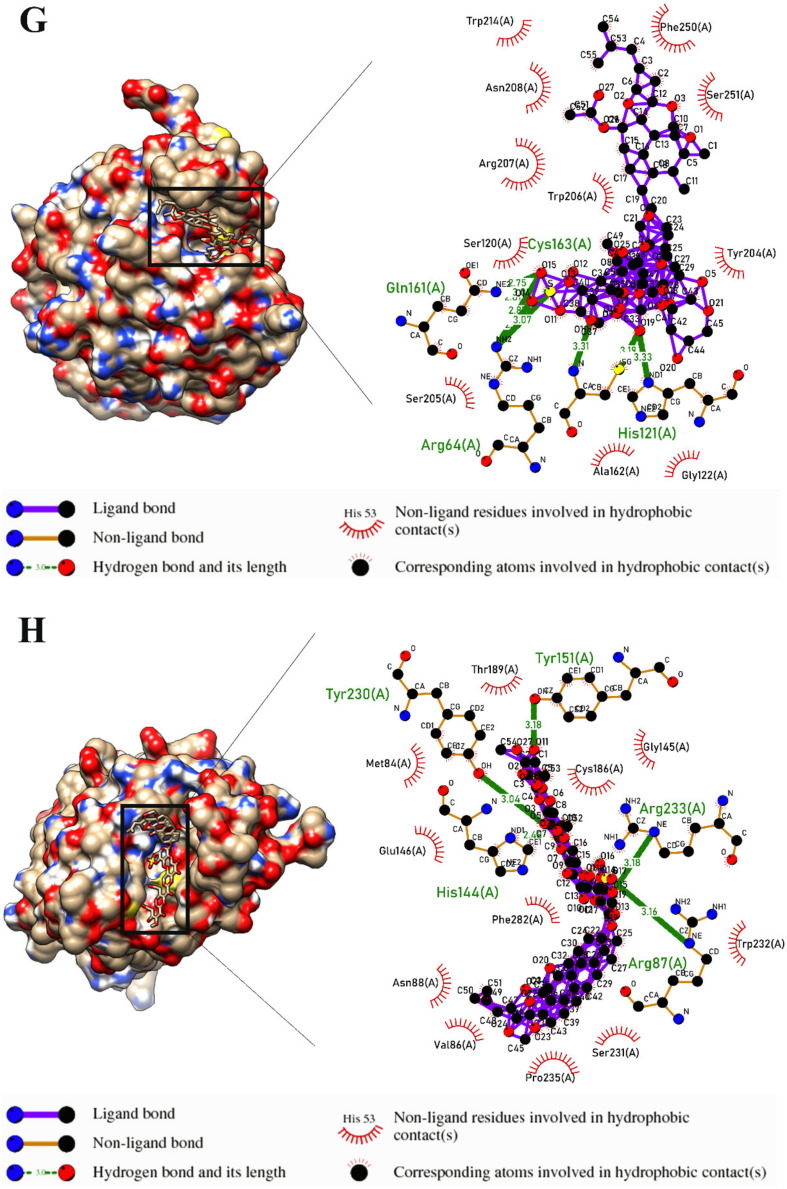

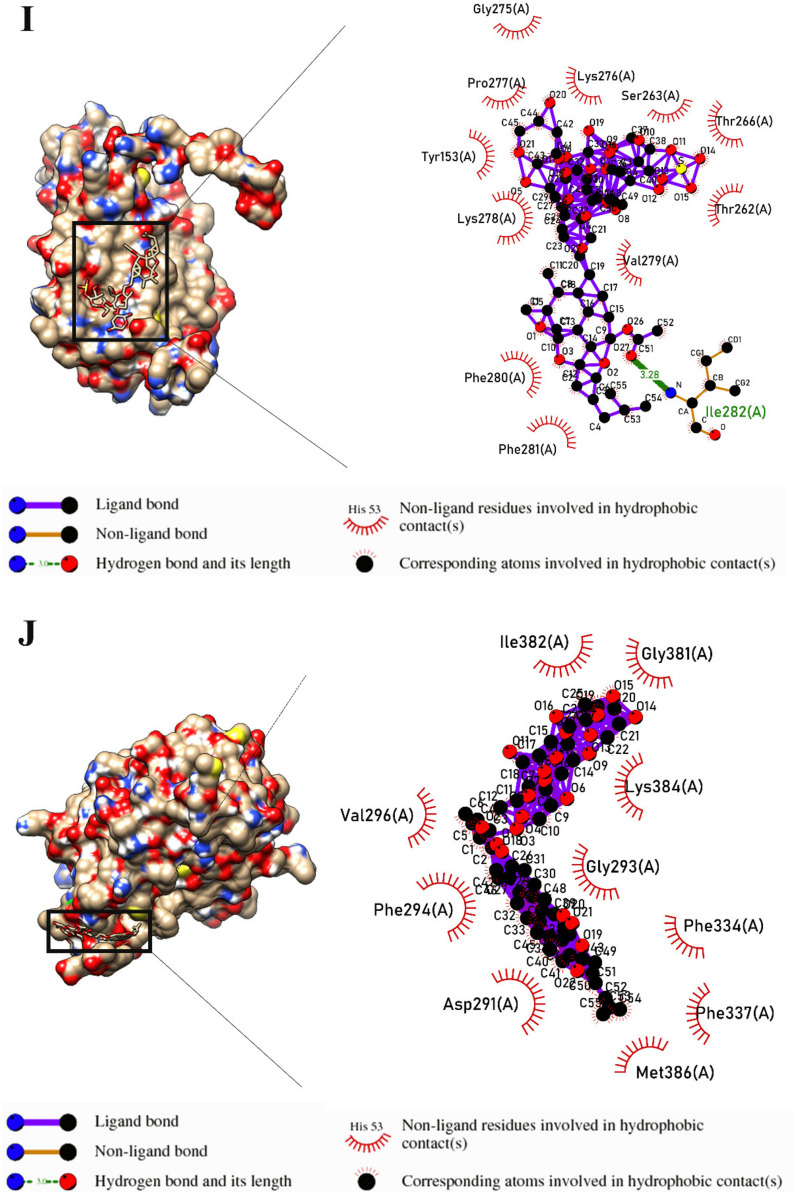

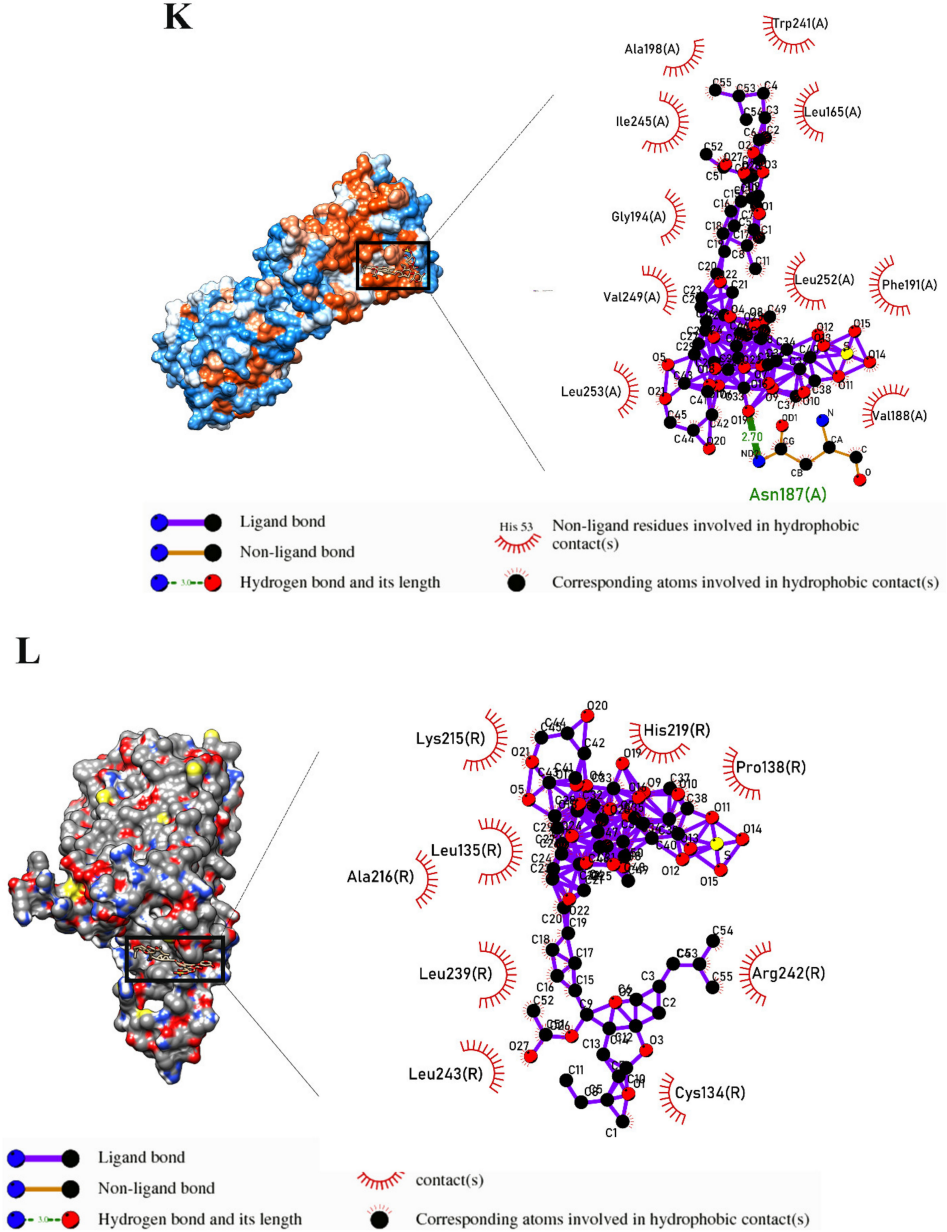

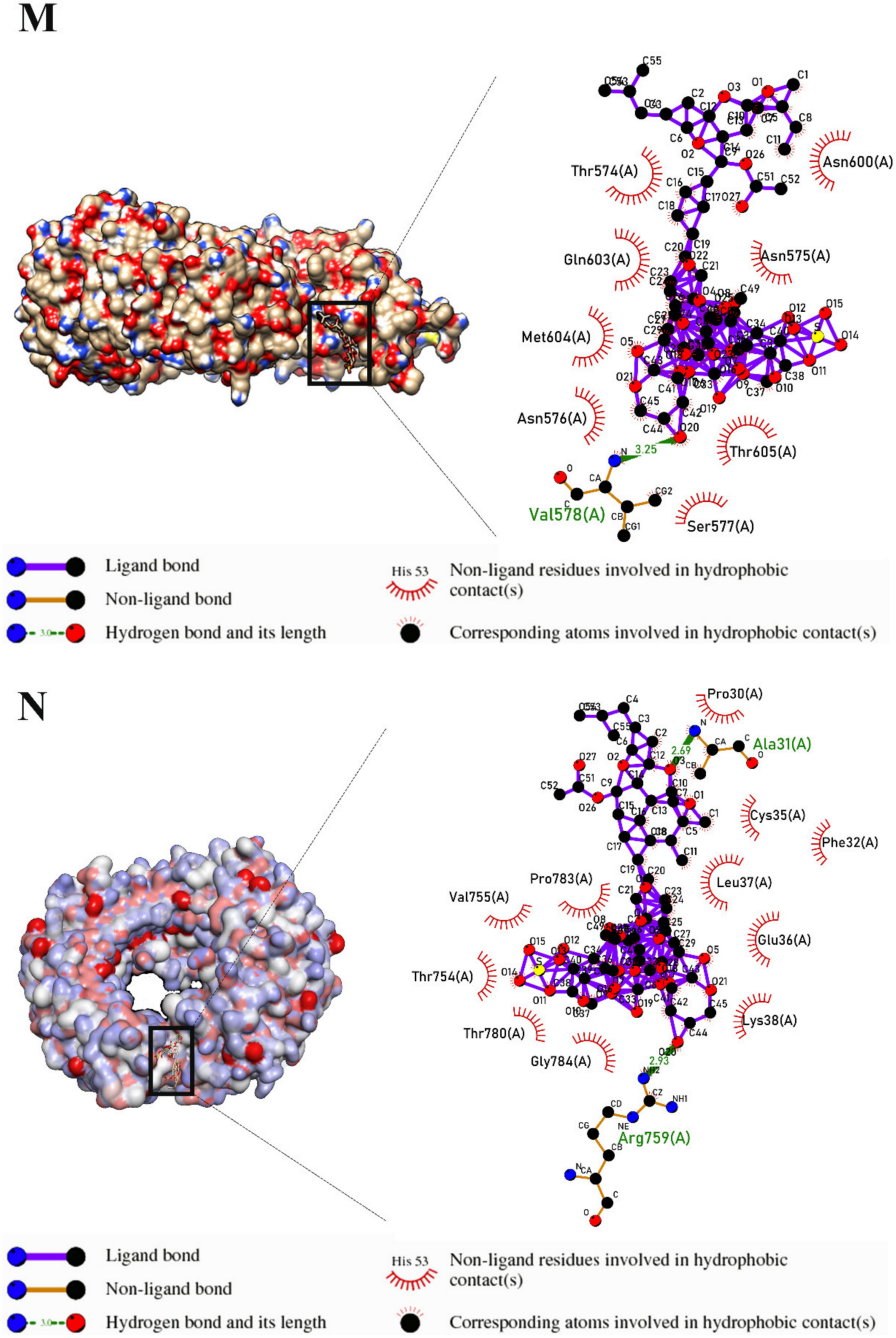

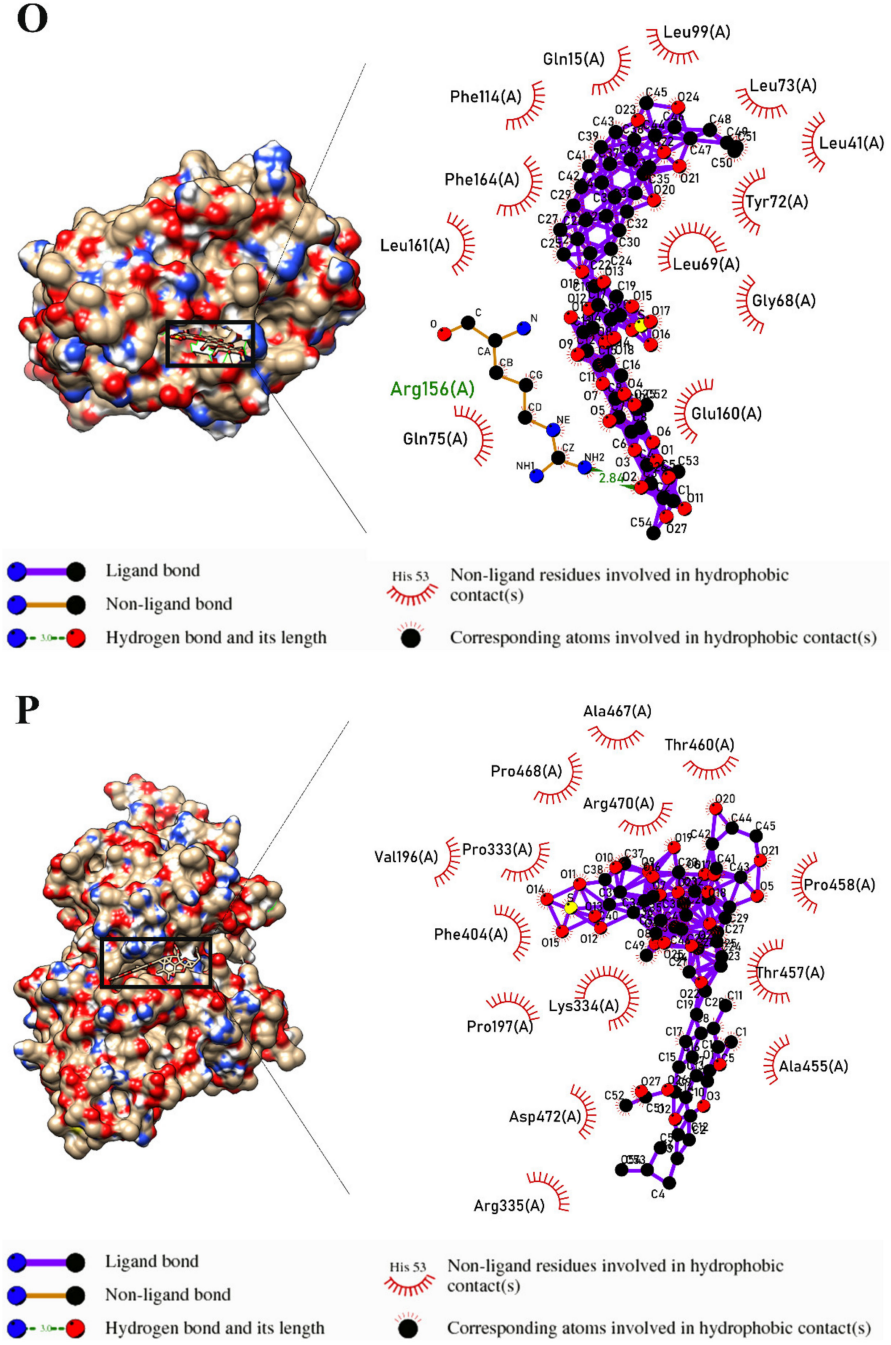

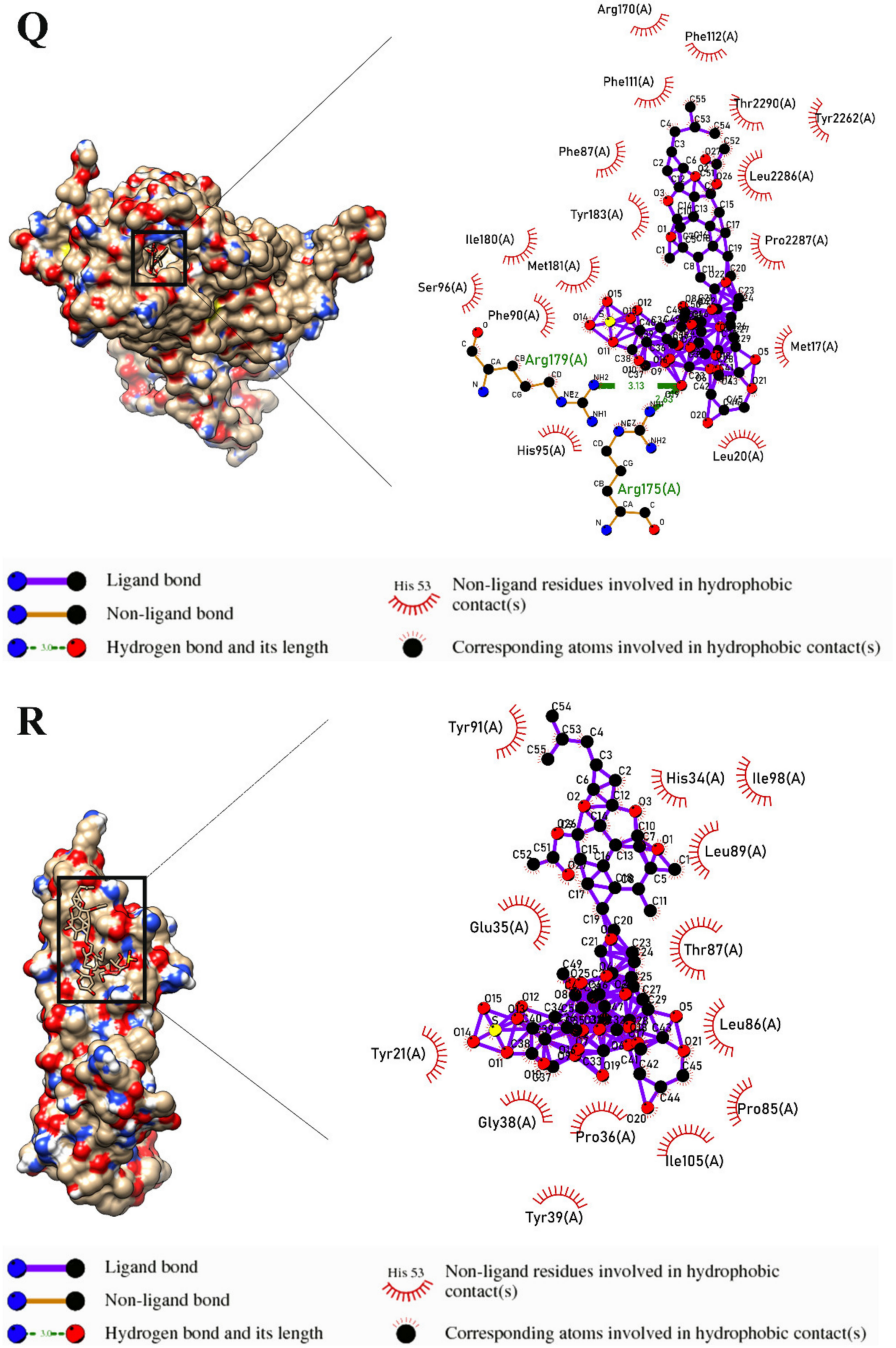
The molecules and interactions that are involved in the binding site of best binding conformation between Intercedenside C, Scabraside A, and Scabraside B and the apoptotic receptors in lung cancer cells. The interactions between Intercedenside C and following receptors: Fas R. (**A**), Tumor necrosis factor receptor 1 (TNFR1) (**B**), Death receptor 4 (DR4) (**C**), Death receptor 5 (DR5) (**D**), Peroxisome proliferator-activated receptor gamma (PPAR-γ) (**F**), Caspase-3 (**G**), Caspase-8 (**I**), Cannabinoid receptor type 1 (CB1) (**K**), Cannabinoid receptor type 2 (CB2) (**L**), Toll-like receptor 4 (TLR- 4) (**M**), Toll-like receptor 9 (TLR- 9) (**N**), Metabotropic glutamate receptor 8 (mGluR 8) (**P**), Prostaglandin D2 (PGD2) (**Q**), Transforming Growth Factor Beta Receptor 2 (TGFBR2) (**R**), Scabraside A with Caspase-9 (**J**), and Scabraside B with the three following receptors: Insulin-like growth factor 1 (IGF-1) (**E**), Caspase-7 (**H**), and Endothelial protein C receptor (EPCR) (**O**) are demonstrated in detail.

**Figure 4:**
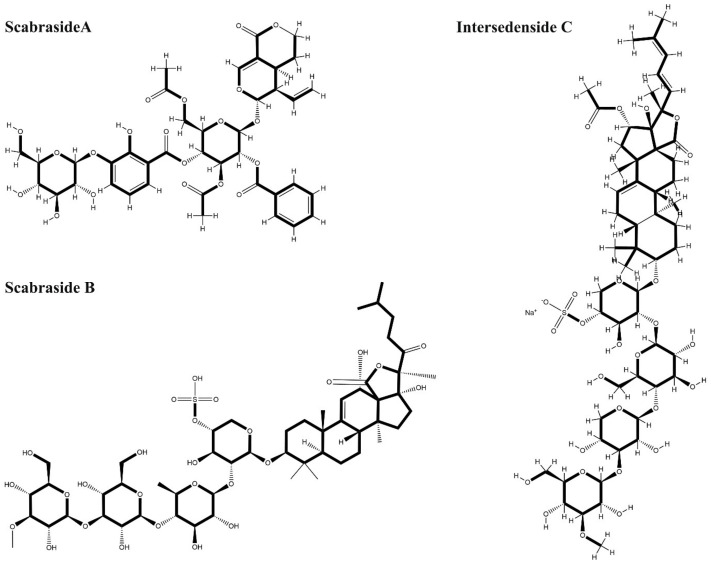
The 2D structure of Intercedenside C and Scabrasides A and B.

Table 3 shows that Intercedenside C exhibited the highest binding affinity to receptors that are involved in the apoptosis process of lung cancer cells, except for Insulin-like growth factor 1 receptor (IGFR1), Caspase-7, Caspase-9, and Endothelial protein C receptor (EPCR). Additionally, Figure 3 displays all the participant molecules and interactions that are involved in the binding process of Intercedenside C with the aforementioned receptors. In contrast, Scabraside B demonstrated the highest affinity to IGFR1, Caspase-7, and EPCR, while Scabraside A exhibited the best tendency towards Caspase-9 (Table 3). The 2D structures of all three components with the highest affinity to the mentioned receptors are shown in Figure 4.

Furthermore, detailed data about binding affinities between all 22 triterpenes and receptors that participated in apoptosis in lung cancer are listed in Table S1 (Supplementary Materials).

## 3. Materials and Methods

### 3.1. Data Collection and Extraction

On 12 February 2023, we performed an accurate assessment on the PubMed online database to identify the most frequent anti-cancer components in the extract of different sorts of sea cucumber using the following search strategy: Search: (sea cucumber[Title/Abstract]) AND ((neoplasm*[Title/Abstract]) OR (neoplasm*[MeSH Terms]) OR (cancer*[MeSH Terms]) OR (cancer*[Title/Abstract]) OR (tumor*[Title/Abstract]) OR (tumor*[MeSH Terms]) OR (cytotoxic*[MeSH Terms]) OR (cytotoxic*[Title/Abstract]) OR (proliferation*[Title/Abstract]) OR (proliferation*[MeSH Terms])).

Next, we analyzed the resulting publications using VOSviewer software (v.1.6.8, 2018) [21], which can analyze the semantic contents of publication titles, keywords, and abstracts, and then relate them to citation count data. The software produced a bubble map that revealed the most frequent compounds that have been studied for their anti-cancer properties in sea cucumber extracts.

We then conducted a search on the Google Scholar database to find anti-lung cancer compounds within the type of component that resulted from the VOSviewer analysis using the following search strategy: (“sea cucumber” “lung cancer” -review -overview). We did not impose any time limitations on either of our search strategies.

### 3.2. Molecular Interactions and Docking Studies of Lung Cancer Apoptotic Pathways and Anti-Lung Cancer Molecules of Sea Cucumber Extract

The 18 most investigated apoptotic receptors were obtained from prior studies [22]. We then utilized AutoDock 4 [23] to examine the binding affinity of all 22 triterpenes in the extracts of various sea cucumbers that have previously shown anti-lung cancer properties against these apoptotic receptors.

We achieved the 3D structures of the 22 ligands and 18 apoptotic receptors (Caspa-se-3, Caspase-7, Caspase-8, Caspase-9, Cannabinoid receptor type 1 (CB1), Cannabinoid receptor type 2 (CB2), Death receptor 4 (DR4), Death receptor 5 (DR5), EPCR, Fas receptor, Insulin-like growth factor 1 receptor (IGF1R), Metabotropic glutamate receptor 8 (mGluR8), Peroxisome proliferator-activated receptor-γ (PPAR-γ), Transforming growth factor beta receptor 2 (TGFBR2), Toll-like receptor 4 (TLR4), Toll-like receptor 9 (TLR9), Tumor necrosis factor receptor 1 (TNFR1), and Prostaglandin D2 (PGD2)) from the PUBCHEM and Protein Data Bank (PDB) databases. The PDB codes for these receptors were 1cp3, 1f1j, 1f9e, 1jxq, 5u09, 6pt0, 5cir, 1za3, 1l8j, 3ezq, 1igr, 6bsz, 1i7i, 4kxz, 2z64, 3wpf, 7k7a, and 6d27, respectively.

In the next step, we omitted non-standard residuals and added hydrogen atoms to each receptor using UCSF Chimera software [24]. We then merged nonpolar hydrogens and ion pairs and assigned Gasteiger partial charges to each ligand atom. Grid boxes were generated using the Computed Atlas of Surface Topography of proteins (CASTp 3.0). Finally, we conducted docking and achieved 10 conformations for each receptor and ligand. All docking conformations were ranked according to the binding affinity and the conformation with the lowest negative energy and RMSD ≤ 2 Å was selected as the best one.

### 3.3. Visualization of Inter-Molecular Interactions

The visualization of the 3D structure of the best conformations was performed using UCSF Chimera software [24]. In addition, the detailed data about intermolecular interactions between the ligand and the receptor were visualized in 2D using LigPlot+ [25].

## 4. Discussion

### 4.1. Sea Cucumbers as an Attractive Anti-Cancer Source

Previous research has shown that sea cucumbers have potential anti-tumor capabilities due to their biologically active components such as fucoidan and chondroitin sulfates [26]. These components have some remarkable activities such as cytotoxic activity, induction of apoptosis and cell cycle arrest, reduction of tumor growth, and anti-metastatic and anti-angiogenic effects. As a result, sea cucumbers have gained the attention of scientists as potential sources of new anti-cancer agents [26]. In the present study, our findings demonstrate that research on the anti-tumor properties of sea cucumbers has been growing in the last few decades (see Figure 1). Our analysis confirms the importance of investigating the anti-cancer properties of sea cucumbers.

### 4.2. Triterpene Glycosides Are the Most Frequently Studied Compounds in the Field of Anti-Tumor Characteristics of Sea Cucumbers

This study revealed that triterpene glycosides have been the most frequently used keyword in studies that are examining the anti-cancer properties of sea cucumbers (see Figure 1 and Table 1). While prior research has already highlighted the significant anti-tumor capabilities of triterpene glycosides [27], the present study emphasizes that these components have been the most commonly used keywords in researches that are exploring the anti-cancer properties of sea cucumbers.

### 4.3. Triterpene Glycosides with the Best Affinity to Apoptotic Receptors in Lung Cancer

#### 4.3.1. Intercedenside C Had the Most Affinity to the Fas Receptor

**Figure 5:**
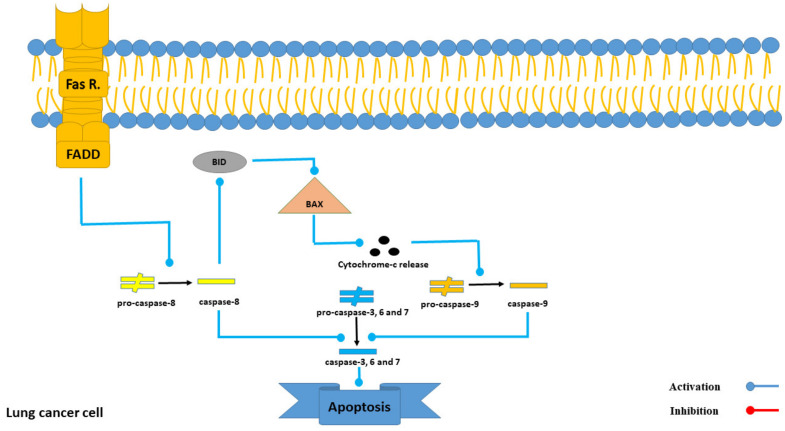
The role of the Fas receptor in the process of apoptosis in lung cancer cells.

Molecular docking analysis revealed that Intercedenside C exhibits the highest affinity to the Fas receptor with a binding affinity of −13.26 Kcal/mole. The Fas receptor is involved in both the intrinsic and extrinsic pathways of apoptosis in lung cancer cells. Upon activation, this receptor triggers the activation of FADD (Fas-associated death domain protein), which subsequently activates Caspase 8 and leads to the apoptosis of target cells [28]. Our results demonstrated that Intercedenside C has the remarkable binding affinity to the Fas receptor in the in silico condition, however, more in vitro and in vivo research is needed to prove this in silico result. Moreover, a detailed mechanism of the Fas receptor’s action in the process of apoptosis in lung cancer cells is illustrated in Figure 5.

#### 4.3.2. Intercedenside C Had the Most Affinity to the TNFR1 Receptor

**Figure 6:**
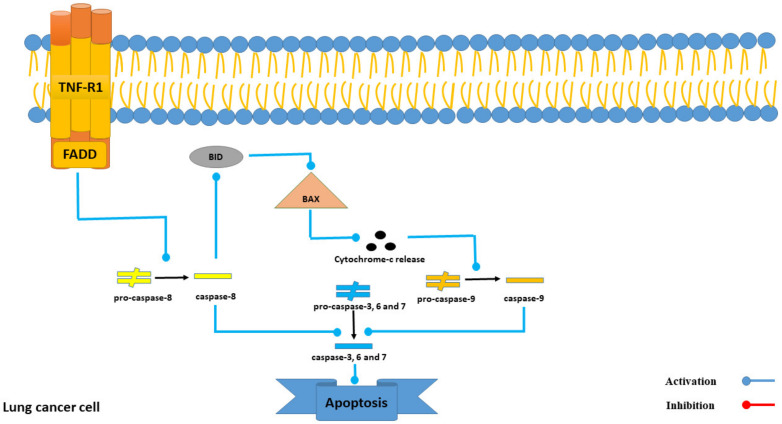
The role of the TNFR1 receptor in the process of apoptosis in lung cancer cells.

Among all mentioned 22 triterpene glycosides in sea cucumber extracts with anti-lung cancer activity (see Table 3), Intercedenside C exhibits the highest binding affinity to TNFR1 with a value of −13.39 Kcal/mole. Based on prior surveys, the activation of TNFR1 leads to the activation of FADD, which subsequently results in apoptosis in lung cancer cells [29]. Notably, despite the high tendency of Intercedenside C towards TNFR1, the need for more in vitro and in vivo surveys in order to examine this tendency exists. Furthermore, the mechanism of TNFR1-induced apoptosis in lung cancer cells is illustrated in Figure 6.

#### 4.3.3. Intercedenside C Had the Most Affinity to the DR4 and DR5 Receptors

**Figure 7:**
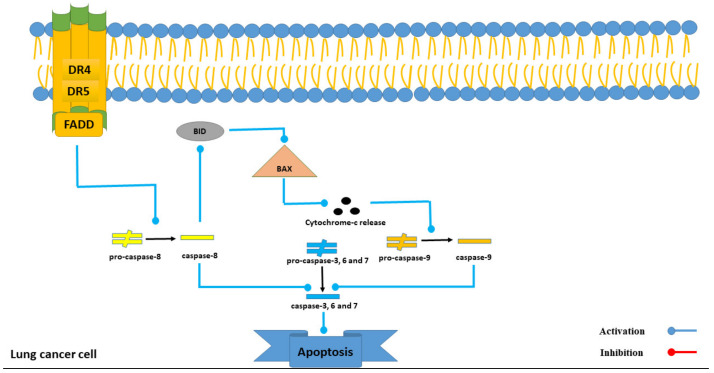
The role of the DR4 and DR5 receptors in the process of apoptosis in lung cancer cells.

Both the DR4 and DR5 receptors are involved in the process of apoptosis in lung cancer cells through the activation of FADD [30]. The detailed mechanism of the induction of apoptosis by these receptors is shown in Figure 7. Additionally, Intercedenside C exhibited the strongest binding affinity towards both the DR4 and DR5 receptors with binding affinity values of −14.98 and −15.35 Kcal/mole, respectively. Although, this in silico finding is consistent with previous studies on the anti-lung cancer activity of Intercedenside C [11], there is still insufficient in vitro and in vivo surveys to confirm the remarkable finding still exists.

#### 4.3.4. Scabraside B Had the Most Affinity to the IGFR1 Receptor

IGFR1 plays a crucial role in the survival, metastasis, and drug resistance of lung cancer cells, making it a valuable therapeutic target for the treatment of this type of cancer [31]. On the other hand, Scabraside B exhibited the strongest binding affinity to this receptor with a binding energy of −15.64 Kcal/mole. However, previous studies have also reported that Scabraside B has anti-cancer effects on lung cancer cells [16]. This suggests that, despite its high affinity for IGFR1 in in silico conditions, Scabraside B may exert its cytotoxic effects on lung cancer cells through other tumor cell-killing pathways, including receptors that induce apoptosis in lung cancer cells.

#### 4.3.5. Intercedenside C Had the Most Affinity to the PPAR-γ Receptor

**Figure 8:**
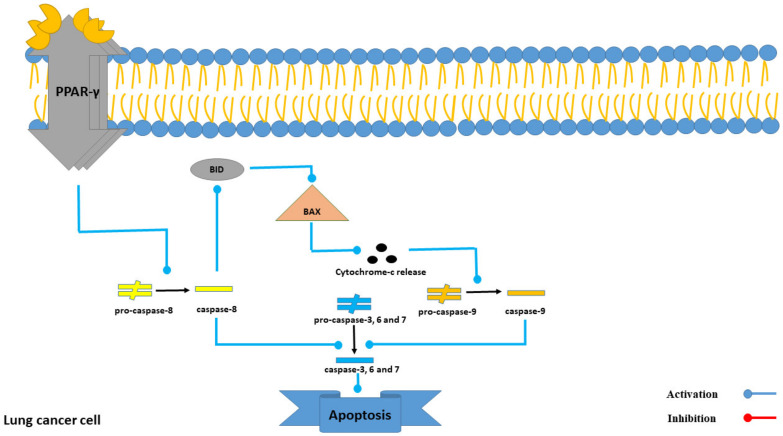
The role of the PPAR-γ receptor in the process of apoptosis in lung cancer cells.

Intercedenside C has exhibited a high affinity to the PPAR-γ receptor among all the 22reported triterpene glycosides (Table 3). PPAR-γ ligands can induce apoptosis through both PPAR-γ-dependent and -independent pathways. Intercedenside C exerts its apoptotic function through the independent pathway [32]. In this pathway, PPAR-γ activation ultimately leads to the activation of a cascade of caspases and induces apoptosis in lung cancer cells [33]. Besides, it is important to know that in spite of high in silico affinity of Intercedenside C to this receptor, more studies are essential (in both in vitro and in vivo conditions) in order to validate this in silico finding. Besides, a detailed mechanism of the induction of apoptosis through the PPAR-γ receptor is shown in Figure 8.

#### 4.3.6. Intercedenside C, Scabraside A, and Scabraside B Had the Most Affinity to Caspases 3, 7, 8, and 9

Caspases 3, 7, 8, and 9 play a crucial role in the process of apoptosis in lung cancer cells. Previous studies have demonstrated that one of the significant mechanisms of targeting lung tumor cells is by activating the mentioned caspases [34]. Therefore, Intercedenside C, Scabraside A, and Scabraside B, with the highest affinity to the aforementioned Caspases, can be considered as potential anti-lung cancer compounds in the structure of sea cucumbers (Table 3). These findings are in accordance with previous studies that have displayed the anti-lung cancer capabilities of all three mentioned components [11,16].

#### 4.3.7. Intercedenside C Had the Most Affinity to CB1 and CB2 Receptors

**Figure 9:**
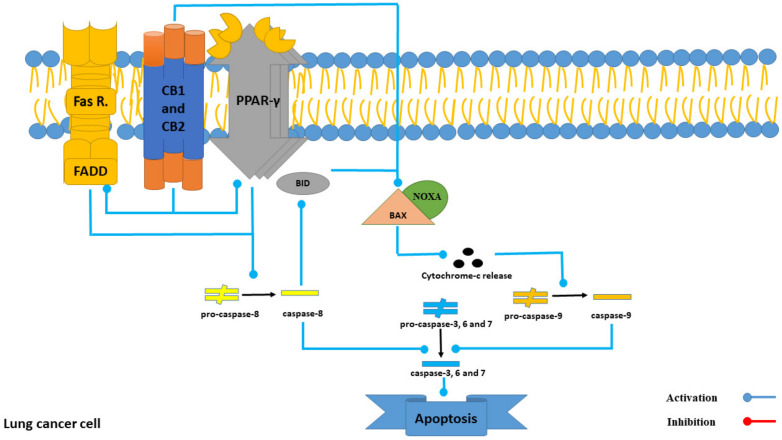
The role of CB1 and CB2 receptor in the process of apoptosis in lung cancer cells.

Both CB1 and CB2 receptors are involved in inducing apoptosis through several mechanisms, including the activation of Fas/FasL, activation of PPARγ, downregulation of Bcl-2, and upregulation of Noxa [35]. On the other hand, Intercedenside C displayed the highest binding affinity to both receptors, with binding energies of −10.61 and −11.86 Kcal/mole, respectively. Thus, our in silico examination demonstrates the tendency of the abovementioned compounds to bind to the mentioned receptor but the need for more research (in both in vitro and in vivo conditions) in order to prove this affinity still exists. Moreover, detailed roles of both receptors in the apoptotic pathway in lung cancer cells are displayed in Figure 9.

#### 4.3.8. Intercedenside C Had the Most Affinity to the TLR4 Receptor

Activation of TLR4 is known to promote survival in lung cancer cells via the PI3K/Akt pathway [36]. Interestingly, Intercedenside C showed the highest binding affinity to this receptor, with a binding energy of −15.64 kcal/mol. However, previous studies have also shown the anti-cancer effects of this triterpene glycoside against lung cancer [11]. Thus, this finding is in opposition to previous research. On the other hand, more in vitro and in vivo studies are required in order to confirm this result.

#### 4.3.9. Intercedenside C Had the Most Affinity to the TLR9 Receptor

In this study, Intercedenside C exhibited the highest affinity to TLR9 among all 22 triterpene glycosides (Table 3). Previous research has also shown that TLR9 activation in lung cancer cells can increase the expression of BAX and P53 [37]. BAX plays a critical role in mitochondrial cell death [38], while P53 plays a crucial role in inducing apoptosis [39]. Therefore, the anti-lung cancer activity of Intercedenside C [11] is in line with the present findings, which demonstrate its high affinity to TLR9.

#### 4.3.10. Scabraside B Had the Most Affinity to the EPCR

Prior studies have indicated that EPCR promotes cell survival in lung cancer cells [40]. However, the present study showed that Scabraside B has the highest affinity to the mentioned receptor (Table 3). Moreover, previous studies have confirmed the anti-lung cancer properties of Scabraside B [16]. Thus, the anti-lung cancer effects of Scabraside B oppose its tendency towards EPCR in the in silico condition, and the affinity of this compound towards receptors that trigger apoptosis in lung cancer cells is more dominant.

#### 4.3.11. Intercedenside C Had the Most Affinity to the mGluR8 Receptor

Previous studies have mentioned that mGluR8 activation induces apoptosis in lung cancer cells [41]. This study has shown that Intercedenside C has the highest binding affinity to the mentioned receptor (Table 3). Thus, this finding confirms the results of previous studies on the anti-lung cancer properties of Intercedenside C [11].

#### 4.3.12. Intercedenside C Had the Most Affinity to the PGD2 Receptor

PGD2 can induce apoptosis in lung cancer cells [42]. On the other hand, among all 22 triterpene glycosides, Intercedenside C displayed the highest affinity towards this receptor with a binding energy of −13.68 Kcal/mole. This result is consistent with the findings of other studies that have confirmed the anti-lung cancer characteristics of Intercedenside C [11].

#### 4.3.13. Intercedenside C Had the Most Affinity to the TGFBR2 Receptor

The TGFBR2 receptor has been an important target for anti-lung cancer agents. Prior studies have shown that inhibiting this receptor can lead to the proliferation and growth of lung cancer cells [43]. Thus, the activation of this receptor may play an important role in tumor suppression in lung cancer. On the other hand, Intercedenside C demonstrated the highest affinity to this receptor (Table 3). This result confirms the findings of other studies on the anti-lung cancer properties of Intercedenside C [11].

### 4.4. Triterpene Glycosides with the Lowest Affinity to Apoptotic Receptors in Lung Cancer

Arguside B is a type of triterpene glycoside and previous studies have demonstrated its anti-lung cancer activity [12]. Despite this fact, our study showed that this compound had the lowest affinity to apoptotic receptors (Table 3). This finding is in contrast to previous studies about the anti-lung cancer capabilities of Arguside B, and this triterpene glycoside may exert its anti-lung tumor impacts through other cellular death pathways.

Another component with the least amount of binding affinity energy was 24-Dehydroechinoside A. This triterpene glycoside is present in the extract of *Holothuria scabra* and has displayed cytotoxic effects in A549 cell lines [16]. Thus, it can be said that its anti-lung cancer capabilities may be mainly due to activating other mechanisms of cell death, not only apoptosis.

### 4.5. Future Insights for the Anti-Lung Cancer Properties of Triterpene Glycosides

Although previous studies have tried to elucidate the anti-cancer mechanisms of sea cucumber compounds [26], the exact anti-cancer mechanisms of the majority of triterpene glycosides are still unclear. In better words, some surveys have tried to explain the anti-cancer mechanisms of triterpene glycosides and reveal some anti-tumor aspects of these components, such as a Caspase-dependent mechanism [44], but the most studied member of the triterpene glycosides is Frondoside A, whose different anti-cancer features have been explained by previous surveys [45]. Although some aspects of the role of Frondoside A in anti-lung cancer pathways have been explained before [44], there is a limitation in the number of surveys in which the anti-lung cancer mechanisms of Intercedenside C, Scabraside A, and Scabraside B have been examined accurately. In other words, previous surveys have only displayed the cytotoxic activity of mentioned compounds [11,16] (Table 2). However, to the best of our knowledge, the main anti-cancer mechanism(s) of these components is still unclear. Thus, more development of methods in order to isolate and purify individual compounds, more studies about their medicinal values, and more in vivo studies are required in order to clarify the accurate anti-cancer pathways in which remarked triterpene glycosides play a crucial part [46].

## 5. Conclusions

This study has demonstrated that sea cucumbers have been of interest to scientists since 1973 for the discovery and development of new anti-cancer components. Moreover, this survey has shown that triterpene glycosides are the most utilized keywords in research in which the anti-tumor properties of sea cucumber have been examined. Furthermore, our study has shown that triterpene glycosides in sea cucumber extracts have a high potential to suppress lung cancer cells. Moreover, among all the triterpene glycosides with anti-lung cancer capabilities, Intercedenside C demonstrated the most affinity to the majority of apoptotic receptors. This result makes Intercedenside C a potent agent against lung cancer, and it may be used in order to produce new anti-lung cancer agents in the near future. Ultimately, an important result from this paper is that in vitro and in vivo studies seem to be crucial in order to discover the accurate mechanisms of the anti-cancer properties of the components of sea cucumber. In summary, this study provides some valuable insights into the potential of sea cucumbers and their triterpene glycosides as a source of anti-cancer agents, particularly for lung cancer.

## Figures and Tables

**Table 1:** Three most frequent keywords in surveys in which the anti-cancer features of sea cucumber are examined.

Keywords	Cluster	Link	Total Link Strength	Occurrence
Triterpene glycosides	12	46	66	24
Fucosylated chondroitin sulfate	18	50	55	14
Frondoside A	13	27	28	9

**Table 2:** The list of triterpene glycosides in the extract of various sea cucumbers with anti-lung cancer properties.

Compounds	Sources	Type of Cancer cell	Mechanism	References
Frondoside A	*Cucumaria frondosa* *Okinawa propolis*	A549 LNM35 NCI-H460-Luc2	Anti-proliferation Anti-metastasis Anti-angiogenesis Anti-invasion Cytotoxicity	[9,10]
Intercedenside A	*Mensamaria intercedens*	Lewis lung carcinoma cells	Cytotoxicity	[11]
Intercedenside B				
Intercedenside C				
Arguside B Arguside C Arguside D Arguside E	*Bohadschia argus*	A549	Cytotoxicity	[12,13,14]
Impatienside A	*Holothuria impatiens*			
Nobiliside D	*Holothuria nobilis*	A549	Cytotoxicity Anti-proliferation Apoptosis	[15]
Scabraside A	*Holothuria scabra*	A549	Cytotoxicity	[16]
Scabraside B				
Scabraside D				[17]
Coloquadranoside A	*Colochirus quadrangularis*	A549	Cytotoxicity	[18]
Philinopside A				
Philinopside B				
Philinopside E				
Pentactaside B	*Pentacta quadrangularis*			[19]
Pentactaside C	*Pentacta quadrangularis*	A549	Cytotoxicity	[19]
Fuscocineroside C	*Holothuria scabra*	A549	Cytotoxicity	[17]
24-Dehydroechinoside A				
Saponin	*Holothuria leucospilota*	A549	Cytotoxicity	[20]

**Table 3:** The affinity of triterpene glycosides with anti-lung cancer properties in the extract of various sea cucumbers with receptors that are involved in the process of apoptosis in lung cancer cells (Kcal/mole).

Compounds	Receptors																	
	Fas R.	TNFR1	DR4	DR5	IGFR1	PPAR-γ	Caspase-3	Caspase-7	Caspase-8	Caspase-9	CB1	CB2	TLR- 4	TLR- 9	EPCR	mGluR 8	PGD2R	TGFBR2
Frondoside A	−6.41	−6.09	−3.93	−5.36	−1.94	−4.71	−4.40	−2.96	−2.25	−6.67	−6.40	−4.38	−5.57	−3.73	−2.82	+1.28	−1.91	−7.08
Intercedenside A	−9.95	−9.34	−12.53	−14.02	−6.93	−10.05	−10.69	−11.32	−12.77	−12.04	−9.66	−10.06	−10.54	−12.93	−12.63	−8.64	−12.47	−12.44
Intercedenside B	−11.98	−9.64	−10.63	−9.66	−8.52	−7.95	−11.26	−11.59	−11.78	−11.94	−10.61	−11.86	−11.80	−11.55	−16.64	−11.10	−13.28	−12.67
Intercedenside C	−13.26	−13.39	−14.98	−15.35	−13.85	−20.23	−19.79	−14.49	−15.14	−16.92	−14.87	−14.04	−15.64	−16.52	−20.14	−13.57	−13.68	−14.16
Arguside B	+0.16	−0.47	−2.06	−0.06	+3.91	+0.94	−1.00	−3.74	+1.71	−0.09	−0.87	−1.65	+2.84	+1.31	−0.92	+1.76	+2.85	−2.67
Arguside C	−4.57	−3.49	−5.91	−2.12	−0.54	−5.08	−6.92	−4.72	−7.40	−5.20	−7.16	−3.63	−4.12	−3.88	−4.32	−2.68	−1.95	−3.21
Arguside D	−3.29	−4.21	−6.99	−8.52	−2.57	−5.11	−3.43	−6.27	−9.09	−6.77	−6.52	−2.96	−7.84	−3.55	−4.67	−4.51	−3.99	−6.57
Arguside E	−4.89	−7.67	−7.09	−5.78	−4.92	−4.48	−4.15	−5.58	−4.97	−5.76	−2.49	−4.45	−3.28	−3.42	−11.42	−4.04	−5.51	−5.82
Impatienside A	−2.14	−3.98	−4.21	−4.50	−0.36	−2.51	−4.52	−3.86	−4.45	−4.55	−3.79	−2.50	−3.61	−3.50	−5.08	−1.37	−2.67	−3.23
Nobiliside D	−9.08	−9.88	−10.42	−9.58	−8.91	−14.75	−11.97	−11.71	−11.36	−9.90	−9.55	−10.69	−9.82	−9.20	−13.07	−8.25	−9.45	−10.14
Scabraside A	−12.35	−13.32	−6.41	−8.01	−12.94	−8.59	−11.18	−14.59	−13.77	−18.04	−9.58	−9.11	−9.80	−5.20	−6.29	−6.87	−6.85	−10.28
Scabraside B	−10.84	−12.97	−13.61	−13.59	−15.64	−15.69	−14.19	−17.94	−12.97	−17.94	−11.07	−13.43	−13.86	−13.05	−20.35	−11.36	−12.34	−12.48
Scabraside D	−7.94	−10.08	−8.08	−8.53	−5.61	−9.03	−8.22	−7.53	−8.49	−8.75	−7.51	−6.02	−4.99	−6.80	−9.11	−6.20	−6.95	−5.08
Coloquadranoside A	−2.00	−4.58	−1.30	−2.60	+1.11	−0.91	−3.65	−4.11	−4.84	−1.78	−3.03	−0.54	−0.85	−0.84	−3.66	−0.74	−1.73	−3.14
Philinopside A	−3.08	−2.19	−4.86	−1.37	−2.19	−4.30	−4.99	−4.77	−5.56	−5.89	−4.79	−5.33	−9.80	−4.07	−6.92	−2.00	−2.08	−6.05
Philinopside B	−4.42	−6.49	−5.78	−5.59	−3.01	−4.11	−7.34	−7.40	−6.29	−7.31	−4.19	−3.78	−1.87	−2.46	−6.01	−3.09	−5.25	−5.75
Philinopside E	−9.33	−9.20	−10.47	−10.47	−10.59	−9.10	−12.18	−13.30	−11.58	−9.37	−9.84	−8.82	−9.11	−9.79	−12.43	−7.88	−9.95	−11.12
Pentactaside B	−9.82	−9.28	−12.55	−9.28	−10.49	−17.17	−11.30	−12.70	−14.13	−11.83	−11.16	−11.41	−9.13	−11.61	−18.02	−8.81	−8.79	−7.52
Pentactaside C	−8.45	−6.76	−7.61	−7.78	−7.06	−6.45	−10.01	−7.82	−6.34	−6.09	−4.07	−9.44	−1.90	−7.44	−4.77	−5.26	−1.93	−9.43
Fuscocineroside C	−6.39	−7.36	−6.30	−6.94	−3.53	−5.50	−6.17	−6.80	−8.11	−7.45	−7.56	−3.64	−5.68	−3.14	−11.77	−4.60	−4.05	−4.71
24-Dehydroechinoside A	+1.25	−0.97	−1.63	+3.96	+0.94	+3.79	−2.06	−0.91	−0.47	−6.85	+0.54	−0.06	+0.89	+0.85	+1.42	+0.73	+1.65	−0.22
Saponin	−5.80	−6.80	−7.49	−5.05	−4.49	−9.25	−4.96	−8.64	−8.78	−8.32	−5.42	−6.92	−5.72	−6.44	−14.98	−3.86	−3.54	−6.95

## Data Availability

Data are contained within the article or Supplementary Materials.

## Supplementary Materials

The following supporting information can be downloaded at: https://www.mdpi.com/article/10.3390/md21050283/s1, Table S1. Detailed information about the binding affinity of triterpene glycosides with anti-lung cancer properties in the extract of sea cucumbers with receptors that are involved in the process of apoptosis in lung cancer cells.

## References

[1] Liu X., Liu Y., Hao J., Zhao X., Lang Y., Fan F., Cai C., Li G., Zhang L., Yu G. (2016). In-vivo anti-cancer mechanism of low-molecular-weight fucosylated chondroitin sulfate (LFCS) from sea cucumber *Cucumaria frondosa*. Molecules.

[2] Gadgeel S.M., Ramalingam S.S., Kalemkerian G.P. (2012). Treatment of lung cancer. Radiol. Clin. N. Am..

[3] Wargasetia T.L., Permana S. (2018). The role of sea cucumber active compound and its derivative as an anti-cancer agent. Curr. Pharmacol. Rep..

[4] Janakiram N.B., Mohammed A., Rao C.V. (2015). Sea cucumbers metabolites as potent anti-cancer agents. Mar. Drugs.

[5] Miri M.R., Zare A., Saberzadeh J., Baghban N., Nabipour I., Tamadon A. (2022). Anti-lung cancer marine compounds: A review. Ther. Innov. Regul. Sci..

[6] Baghban N., Khoradmehr A., Nabipour I., Tamadon A., Ullah M. (2022). The potential of marine-based gold nanomaterials in cancer therapy: A mini-review. Gold Bull..

[7] Hossain A., Dave D., Shahidi F. (2022). Antioxidant potential of sea cucumbers and their beneficial effects on human health. Mar. Drugs.

[8] Ru R., Guo Y., Mao J., Yu Z., Huang W., Cao X., Hu H., Meng M., Yuan L. (2022). Cancer cell inhibiting sea cucumber (*Holothuria leucospilota*) protein as a novel anti-cancer drug. Nutrients.

[9] Nguyen B.C.Q., Yoshimura K., Kumazawa S., Tawata S., Maruta H. (2017). Frondoside A from sea cucumber and nymphaeols from *Okinawa propolis*: Natural anti-cancer agents that selectively inhibit PAK1 In-Vitro. Drug Discov. Ther..

[10] Sajwani F.H. (2019). Frondoside A is a potential anticancer agent from sea cucumbers. J. Cancer Res. Ther..

[11] Zou Z.-R., Yi Y.-H., Wu H.-M., Wu J.-H., Liaw C.-C., Lee K.-H. (2003). Intercedensides A−C, three new cytotoxic triterpene glycosides from the sea cucumber *Mensamaria intercedens* Lampert. J. Nat. Prod..

[12] Liu B.S., Yi Y.H., Li L., Sun P., Yuan W.H., Sun G.Q., Han H., Xue M. (2008). Argusides B and C, two new cytotoxic triterpene glycosides from the sea cucumber *Bohadschia argus* Jaeger. Chem. Biodivers..

[13] Sun P., Liu B.S., Yi Y.H., Li L., Gui M., Tang H.F., Zhang D.Z., Zhang S.L. (2007). A new cytotoxic lanostane-type triterpene glycoside from the sea cucumber *Holothuria impatiens*. Chem. Biodivers..

[14] Liu B.S., Yi Y.H., Li L., Sun P., Han H., Sun G.Q., Wang X.H., Wang Z.L. (2008). Argusides D and E, two new cytotoxic triterpene glycosides from the sea cucumber Bohadschia argus Jaeger. Chem. Biodivers..

[15] Zhang J.-J., Zhu K.-Q. (2017). A novel antitumor compound nobiliside D isolated from sea cucumber (*Holothuria nobilis* Selenka). Exp. Ther. Med..

[16] Han H., Yi Y., Xu Q., La M., Zhang H. (2009). Two new cytotoxic triterpene glycosides from the sea cucumber *Holothuria scabra*. Planta Med..

[17] Hua H., Ling L., Yi Y.-H., Wang X.-H., Pan M.-X. (2012). Triterpene glycosides from sea cucumber *Holothuria scabra* with cytotoxic activity. Chin. Herb. Med..

[18] Yang W.-S., Qi X.-R., Xu Q.-Z., Yuan C.-H., Yi Y.-H., Tang H.-F., Shen L., Han H. (2021). A new sulfated triterpene glycoside from the sea cucumber *Colochirus quadrangularis*, and evaluation of its antifungal, antitumor and immunomodulatory activities. Bioorg. Med. Chem..

[19] Han H., Xu Q.Z., Yi Y.H., Gong W., Jiao B.H. (2010). Two new cytotoxic disulfated holostane glycosides from the sea cucumber *Pentacta quadrangularis*. Chem. Biodivers..

[20] Soltani M., Parivar K., Baharara J., Kerachian M.A., Asili J. (2014). Hemolytic and cytotoxic properties of saponin purified from *Holothuria leucospilota* sea cucumber. Rep. Biochem. Mol. Biol..

[21] Van Eck N., Waltman L. (2010). Software survey: VOSviewer, a computer program for bibliometric mapping. Scientometrics.

[22] Zare A., Afshar A., Khoradmehr A., Baghban N., Mohebbi G., Barmak A., Daneshi A., Bargahi A., Nabipour I., Almasi-Turk S. (2023). Chemical compositions and experimental and computational modeling of the anticancer effects of cnidocyte venoms of jellyfish *Cassiopea andromeda* and *Catostylus mosaicus* on human adenocarcinoma A549 cells. Mar. Drugs.

[23] Morris G.M., Huey R., Lindstrom W., Sanner M.F., Belew R.K., Goodsell D.S., Olson A.J. (2009). AutoDock4 and AutoDockTools4: Automated docking with selective receptor flexibility. J. Comput. Chem..

[24] Pettersen E.F., Goddard T.D., Huang C.C., Couch G.S., Greenblatt D.M., Meng E.C., Ferrin T.E. (2004). UCSF Chimera—A visualization system for exploratory research and analysis. J. Comput. Chem..

[25] Laskowski R.A., Swindells M.B. (2011). LigPlot+: Multiple Ligand–Protein Interaction Diagrams for Drug Discovery.

[26] Wargasetia T.L. (2017). Mechanisms of cancer cell killing by sea cucumber-derived compounds. Investig. New Drugs.

[27] Mondol M.A.M., Shin H.J., Rahman M.A., Islam M.T. (2017). Sea cucumber glycosides: Chemical structures, producing species and important biological properties. Mar. Drugs.

[28] Gordon N., Kleinerman E.S. (2010). Aerosol therapy for the treatment of osteosarcoma lung metastases: Targeting the Fas/FasL pathway and rationale for the use of gemcitabine. J. Aerosol. Med. Pulm. Drug Deliv..

[29] Sanlioglu A.D., Aydin C., Bozcuk H., Terzioglu E., Sanlioglu S. (2004). Fundamental principals of tumor necrosis factor-alpha gene therapy approach and implications for patients with lung carcinoma. Lung Cancer.

[30] Yang A., Wilson N.S., Ashkenazi A. (2010). Proapoptotic DR4 and DR5 signaling in cancer cells: Toward clinical translation. Curr. Opin. Cell Biol..

[31] Velcheti V., Govindan R. (2006). Insulin-like growth factor and lung cancer. J. Thorac. Oncol..

[32] Elrod H.A., Sun S.-Y. (2008). PPARγ and apoptosis in cancer. PPAR Res..

[33] Li M., Lee T.W., Yim A.P., Mok T.S., Chen G.G. (2006). Apoptosis induced by troglitazone is both peroxisome proliferator-activated receptor-γ-and ERK-dependent in human non-small lung cancer cells. J. Cell. Physiol..

[34] Zhang C.-C., Li C.-G., Wang Y.-F., Xu L.-H., He X.-H., Zeng Q.-Z., Zeng C.-Y., Mai F.-Y., Hu B., Ouyang D.-Y. (2019). Chemotherapeutic paclitaxel and cisplatin differentially induce pyroptosis in A549 lung cancer cells via caspase-3/GSDME activation. Apoptosis.

[35] Pagano C., Navarra G., Coppola L., Bifulco M., Laezza C. (2021). Molecular mechanism of cannabinoids in cancer progression. Int. J. Mol. Sci..

[36] Zhang R., Dong Y., Sun M., Wang Y., Cai C., Zeng Y., Wu Y., Zhao Q. (2019). Tumor-associated inflammatory microenvironment in non-small cell lung cancer: Correlation with FGFR1 and TLR4 expression via PI3K/Akt pathway. J. Cancer.

[37] Yuan S., Qiao T., Li X., Zhuang X., Chen W., Chen X., Zhang Q. (2018). Toll-like receptor 9 activation by CpG oligodeoxynucleotide 7909 enhances the radiosensitivity of A549 lung cancer cells via the p53 signaling pathway. Oncol. Lett..

[38] Spitz A.Z., Gavathiotis E. (2022). Physiological and pharmacological modulation of BAX. Trends Pharmacol. Sci..

[39] Liu J., Zhang C., Wang J., Hu W., Feng Z. (2020). The regulation of ferroptosis by tumor suppressor p53 and its pathway. Int. J. Mol. Sci..

[40] Antón I., Molina E., Luis-Ravelo D., Zandueta C., Valencia K., Ormazabal C., Martínez-Canarias S., Perurena N., Pajares M.J., Agorreta J. (2012). Receptor of activated protein C promotes metastasis and correlates with clinical outcome in lung adenocarcinoma. Am. J. Respir. Crit..

[41] Li T.-J., Huang Y.-H., Chen X., Zhou Z., Luo S.-W., Feng D.-D., Han J.-Z., Luo Z.-Q. (2015). Metabotropic glutamate receptor 8 activation promotes the apoptosis of lung carcinoma A549 cells In-Vitro. Sheng Li Xue Bao.

[42] Jara-Gutiérrez Á., Baladrón V. (2021). The role of prostaglandins in different types of cancer. Cells.

[43] Li G., Wu F., Yang H., Deng X., Yuan Y. (2017). MiR-9-5p promotes cell growth and metastasis in non-small cell lung cancer through the repression of TGFBR2. Biomed. Pharmacother..

[44] Aminin D.L., Menchinskaya E.S., Pisliagin E.A., Silchenko A.S., Avilov S.A., Kalinin V.I. (2015). Anticancer activity of sea cucumber triterpene glycosides. Mar. Drugs.

[45] Adrian T.E., Collin P. (2018). The anti-cancer effects of frondoside A. Mar. Drugs.

[46] Sireesha D. (2019). Treatment of cancer by using sea cucumber. Int. J. Res. Eng. Sci. Manag..

