# Supplementary material for: A Bibliometric and In Silico-Based Analysis of Anti-Lung Cancer Compounds from Sea Cucumber
Source: Mar Drugs. 2023 Apr 28;21(5):283. doi: 10.3390/md21050283 (PMC10223633; doi:10.3390/md21050283)
Supplement: Supplementary file 1 [file marinedrugs-21-00283-s001.zip › marinedrugs-2336155-supplementary.pdf]

A bibliometric and *in-silico* based-analysis of anti-lung cancer compounds of sea cucumber

Afshin Zare[1], Mussin Nadiar Maratovich[2], Kaliyev Asset Askerovich[2], Abenova Nurgul Abdullayevna[3], Farhad Rahmanifar[4], Mahdi Mahdipour[5,6], Shabnam Bakhshalizadeh[7,8], Reza Shirazi[9], Nader Tanideh[1,10,11] and Amin Tamadon[1,12*]

[1] The PerciaVista Biotechnology Company, Shiraz, Iran. afshinzareresearch@gmail.com (A.Z.); amintamaddon@yahoo.com (A.T.)
[2] General Surgery, West Kazakhstan Marat Ospanov Medical University, Aktobe, Kazakhstan. nadiar_musin@zkmu.kz (M.N.M.); aset_kaliyev@mail.ru (K.A.A.)
[3] Department of Internal Diseases, West Kazakhstan Marat Ospanov Medical University, Aktobe, Kazakhstan. nurgul.abenova@gmail.com (A.N.A.)
[4] Department of Basic Sciences, School of Veterinary Medicine, Shiraz University, Shiraz, Iran. f.rahmanifar@yahoo.com (F.R.)
[5] Stem Cell Research Center, Tabriz University of Medical Sciences, Tabriz, Iran. mahdi.mahdipour@gmail.com (M.M.)
[6] Department of Applied Cell Sciences, Faculty of Advanced Medical Sciences, Tabriz University of Medical Sciences, Tabriz, Iran
[7] Reproductive Development, Murdoch Children's Research Institute, Melbourne, Victoria, Australia. bakhshalizadehshabnam@gmail.com (S.B.)
[8] Department of Paediatrics, University of Melbourne, Melbourne, Victoria, Australia.
[9] Department of Anatomy, School of Medical Sciences, Biomedical & Health, UNSW Sydney, Sydney, Australia. reza.shirazi@unsw.edu.au (R.S.)
[10] Stem Cells Technology Research Center, Shiraz University of Medical Sciences, 73, Shiraz, Iran. tanidehn@gmail.com (N.T.)
[11] Department of Pharmacology, Medical School, Shiraz University of Medical Sciences, Shiraz, 73, Iran
[12] Department for Scientific Work, West Kazakhstan Marat Ospanov Medical University, Aktobe, Kazakhstan. amintamaddon@yahoo.com (A.T.)
* Correspondence: amintamaddon@yahoo.com; Tel/fax: +7 705 629 9350 (A.T.)

Table S1. Detailed information about binding affinity of triterpene glycosides with anti-lung cancer properties in the extract of sea cucumbers with receptors that are involve in the process of apoptosis in lung cancer cells

Receptor	Ligand	RMSD	Binding energy (kcal/mol)	Inhibition constant (Ki)
Fas R.	Frondoside A	92.61	-6.41	19.91 uM
	Intercedenside A	96.82	-9.95	51.18 nM
	Intercedenside B	80.37	-11.98	1.65 nM
	Intercedenside C	91.53	-13.26	189.83 pM
	Arguside B	80.37	+0.16	NA
	Arguside C	93.87	-4.57	449.53 uM
	Arguside D	97.70	-3.29	3.88 mM
	Arguside E	86.50	-4.89	259.03 uM
	Impatienside A	92.50	-2.14	27.13 mM
	Nobiliside D	82.02	-9.08	220.17 nM
	Scabraside A	75.36	-12.35	885.04 pM
	Scabraside B	89.27	-10.84	11.41 nM
	Scabraside D	93.78	-7.94	1.51 uM
	Coloquadranoside A	85.75	-2.00	34.46 mM
	Philinopside A	90.05	-3.08	5.50 mM
	Philinopside B	90.58	-4.42	572.28 uM
	Philinopside E	84.27	-9.33	145.45 nM
	Pentactaside B	96.64	-9.82	63.22 nM
	Pentactaside C	80.72	-8.45	638.67 nM
	Fuscocineroside C	92.38	-6.39	20.88 uM
	24-Dehydroechinoside A	97.03	+1.25	NA
	Saponin	88.98	-5.80	56.12 uM
TNFR1	Frondoside A	13.46	-6.09	34.24 uM
	Intercedenside A	29.37	-9.34	141.51 nM
	Intercedenside B	23.22	-9.64	85.71 nM
	Intercedenside C	23.28	-13.39	153.57 pM
	Arguside B	18.02	-0.47	453.89mM
	Arguside C	21.97	-3.49	2.76 mM
	Arguside D	15.68	-4.21	820.17 uM
	Arguside E	23.32	-7.67	2.37 uM
	Impatienside A	12.62	-3.98	1.22 mM
	Nobiliside D	28.96	-9.88	57.72 nM
	Scabraside A	16.46	-13.32	171.62 pM

	Scabraside B	21.09	-12.97	311.62 pM
	Scabraside D	23.14	-10.08	40.94 nM
	Coloquadranoside A	17.35	-4.58	441.64 uM
	Philinopside A	66.29	-2.19	24.69 mM
	Philinopside B	3.74	-6.49	17.40 uM
	Philinopside E	27.46	-9.20	178.95 nM
	Pentactaside B	28.33	-9.28	158.29 nM
	Pentactaside C	8.79	-6.76	11.03 uM
	Fuscocineroside C	24.69	-7.36	4.03 uM
	24-Dehydroechinoside A	23.75	-0.97	193.05 mM
	Saponin	21.32	-6.80	10.35 uM
DR4	Frondoside A	20.57	-3.93	1.31mM
	Intercedenside A	26.50	-12.53	652.84 pM
	Intercedenside B	42.30	-10.63	16.28 nM
	Intercedenside C	35.73	-14.98	10.42 pM
	Arguside B	33.92	-2.06	30.94 mM
	Arguside C	30.62	-5.91	46.74 uM
	Arguside D	30.22	-6.99	7.53 uM
	Arguside E	34.15	-7.09	6.32 uM
	Impatienside A	32.43	-4.21	824.83 uM
	Nobiliside D	32.11	-10.42	22.88 nM
	Scabraside A	29.54	-6.41	20.10 uM
	Scabraside B	35.23	-13.61	105.65 pM
	Scabraside D	33.46	-8.08	1.19 uM
	Coloquadranoside A	47.67	-1.30	111.39 mM
	Philinopside A	38.65	-4.86	272.24 uM
	Philinopside B	39.39	-5.78	58.24 uM
	Philinopside E	42.94	-10.47	20.99 nM
	Pentactaside B	33.35	-12.55	628.98 pM
	Pentactaside C	36.00	-7.61	2.62 uM
	Fuscocineroside C	27.84	-6.30	24.25 uM
	24-Dehydroechinoside A	41.22	-1.63	63.55 mM
	Saponin	30.79	-7.49	3.22 uM

DR5	Frondoside A	61.63	-5.36	118.67 uM
	Intercedenside A	69.10	-14.02	52.96 pM
	Intercedenside B	60.79	-9.66	82.86 nM
	Intercedenside C	61.09	-15.35	5.65 pM
	Arguside B	62.69	-0.06	904.79 mM
	Arguside C	58.59	-2.12	27.90 mM
	Arguside D	66.01	-8.52	565.63 nM
	Arguside E	62.15	-5.78	58.21 uM
	Impatienside A	64.77	-4.50	505.27 uM
	Nobiliside D	66.82	-9.58	94.72 nM
	Scabraside A	65.11	-8.01	1.35 uM
	Scabraside B	57.13	-13.59	108.79 pM
	Scabraside D	69.92	-8.53	559.84 nM
	Coloquadranoside A	55.10	-2.60	12.51 mM
	Philinopside A	63.71	-1.37	99.41 mM
	Philinopside B	59.44	-5.59	79.38 uM
	Philinopside E	61.26	-10.47	20.98 nM
	Pentactaside B	61.08	-9.28	157.70 nM
	Pentactaside C	59.59	-7.78	1.99 uM
	Fuscocineroside C	60.13	-6.94	8.15 uM
	24-Dehydroechinoside A	40.66	+3.96	NA
	Saponin	55.36	-5.05	198.71 uM
IGFR1	Frondoside A	54.50	-1.94	37.66 mM
	Intercedenside A	76.40	-6.93	8.36 uM
	Intercedenside B	69.73	-8.52	569.59 nM
	Intercedenside C	52.40	-13.85	70.14 pM
	Arguside B	43.73	+3.91	NA
	Arguside C	72.48	-0.54	402.45 mM
	Arguside D	91.02	-2.57	12.96 mM
	Arguside E	58.18	-4.92	248.36 uM
	Impatienside A	55.33	-0.36	542.48 mM
	Nobiliside D	58.12	-8.91	293.21 nM
	Scabraside A	81.09	-12.94	328.70 pM

	Scabraside B	64.28	-15.64	3.41 pM
	Scabraside D	49.68	-5.61	77.58 uM
	Coloquadranoside A	55.68	+1.11	NA
	Philinopside A	66.29	-2.19	24.69 mM
	Philinopside B	57.75	-3.01	6.19 mM
	Philinopside E	79.71	-10.59	17.17 nM
	Pentactaside B	79.24	-10.49	20.32 nM
	Pentactaside C	61.68	-7.06	6.71 uM
	Fuscocineroside C	67.67	-3.53	2.59 mM
	24-Dehydroechinoside A	98.19	+0.94	NA
	Saponin	69.98	-4.49	513.36 uM
PPAR-γ	Frondoside A	35.64	-4.71	354.95 uM
	Intercedenside A	32.37	-10.05	43.02 nM
	Intercedenside B	52.32	-7.95	1.49 uM
	Intercedenside C	26.88	-20.23	1.47 fM
	Arguside B	25.09	+0.94	NA
	Arguside C	4.91	-5.08	189.93 uM
	Arguside D	14.97	-5.11	180.81 uM
	Arguside E	31.83	-4.48	516.69 uM
	Impatienside A	38.60	-2.51	14.42 mM
	Nobiliside D	25.66	-14.75	15.33 pM
	Scabraside A	30.76	-8.59	508.16 nM
	Scabraside B	24.14	-15.69	3.17 pM
	Scabraside D	22.13	-9.03	241.35 nM
	Coloquadranoside A	15.05	-0.91	216.98 mM
	Philinopside A	11.12	-4.30	703.49 uM
	Philinopside B	34.12	-4.11	963.21 uM
	Philinopside E	31.56	-9.10	213.28 nM
	Pentactaside B	28.70	-17.17	258.77 fM
	Pentactaside C	12.20	-6.45	18.58 uM
	Fuscocineroside C	18.82	-5.50	92.31 uM
	24-Dehydroechinoside A	8.43	+3.79	NA
	Saponin	23.59	-9.25	165.34 nM

Caspase-3	Frondoside A	21.82	-4.40	591.23 uM
	Intercedenside A	34.03	-10.69	14.47 nM
	Intercedenside B	44.05	-11.26	5.57 nM
	Intercedenside C	44.27	-19.79	3.14 fM
	Arguside B	55.10	-1.00	185.05 mM
	Arguside C	43.91	-6.92	8.47 uM
	Arguside D	33.74	-3.43	3.07 mM
	Arguside E	41.61	-4.15	902.11 uM
	Impatienside A	24.23	-4.52	489.96 uM
	Nobiliside D	48.36	-11.97	1.69 nM
	Scabraside A	41.82	-11.18	6.39 nM
	Scabraside B	40.76	-14.19	39.49 pM
	Scabraside D	19.16	-8.22	942.64 nM
	Coloquadranoside A	13.46	-3.65	2.13 mM
	Philinopside A	25.45	-4.99	220.88 uM
	Philinopside B	46.61	-7.34	4.14 uM
	Philinopside E	44.20	-12.18	1.18 nM
	Pentactaside B	51.74	-11.30	5.24 nM
	Pentactaside C	49.92	-10.01	46.25 nM
	Fuscocineroside C	48.92	-6.17	30.19 uM
	24-Dehydroechinoside A	27.81	-2.06	30.71 mM
	Saponin	49.51	-4.96	232.85 uM
Caspase-7	Frondoside A	20.71	-2.96	6.76 mM
	Intercedenside A	38.39	-11.32	5.07 nM
	Intercedenside B	31.79	-11.59	3.17 nM
	Intercedenside C	32.53	-14.49	23.88 pM
	Arguside B	54.18	-3.74	1.81 mM
	Arguside C	53.26	-4.72	347.78 uM
	Arguside D	52.81	-6.27	25.15 uM
	Arguside E	27.20	-5.58	81.27 uM
	Impatienside A	55.53	-3.86	1.49 mM
	Nobiliside D	23.74	-11.71	2.60 nM
	Scabraside A	30.21	-14.59	20.33 pM

	Scabraside B	26.92	-15.42	4.99 pM
	Scabraside D	25.05	-7.53	3.03 uM
	Coloquadranoside A	39.36	-4.11	971.58 uM
	Philinopside A	45.87	-4.77	317.54 uM
	Philinopside B	37.58	-7.40	3.75 uM
	Philinopside E	24.81	-13.30	177.39 pM
	Pentactaside B	42.31	-12.70	494.51 pM
	Pentactaside C	32.72	-7.82	1.85 uM
	Fuscocineroside C	31.69	-6.80	10.36 uM
	24-Dehydroechinoside A	60.13	-0.91	216.41 mM
	Saponin	63.41	-8.64	464.29 nM
Caspase-8	Frondoside A	79.59	-2.25	22.47 mM
	Intercedenside A	73.53	-8.93	284.07 nM
	Intercedenside B	67.97	-11.78	2.32 nM
	Intercedenside C	69.22	-15.14	7.94 pM
	Arguside B	42.66	+1.71	NA
	Arguside C	54.56	-7.40	3.76 uM
	Arguside D	62.08	-9.09	218.75 nM
	Arguside E	63.95	-4.97	228.46 uM
	Impatienside A	54.82	-4.45	550.69 uM
	Nobiliside D	79.40	-11.36	4.70 nM
	Scabraside A	60.11	-13.77	80.39 pM
	Scabraside B	57.59	-12.97	310.92 pM
	Scabraside D	54.45	-8.49	596.64 nM
	Coloquadranoside A	50.72	-4.84	281.67 uM
	Philinopside A	54.65	-5.56	84.65 uM
	Philinopside B	58.90	-6.29	24.45 uM
	Philinopside E	69.83	-11.58	3.22 nM
	Pentactaside B	71.34	-14.13	44.23 pM
	Pentactaside C	86.09	-6.34	22.63 uM
	Fuscocineroside C	60.44	-8.11	1.13 uM
	24-Dehydroechinoside A	67.43	-0.47	449.58 mM
	Saponin	62.45	-8.78	368.79 nM

Caspase-9	Frondoside A	45.89	-6.67	12.89 uM
	Intercedenside A	58.18	-12.04	1.49 nM
	Intercedenside B	54.76	-11.94	1.76 nM
	Intercedenside C	48.83	-16.92	396.76 fM
	Arguside B	49.80	-0.09	852.70 mM
	Arguside C	30.00	-5.20	153.06 uM
	Arguside D	29.32	-6.77	10.97 uM
	Arguside E	26.69	-5.76	60.13 uM
	Impatienside A	40.39	-4.55	459.85 uM
	Nobiliside D	33.73	-9.90	55.50 nM
	Scabraside A	35.22	-18.04	59.77 fM
	Scabraside B	53.13	-17.94	70.63 fM
	Scabraside D	40.47	-8.75	382.67 nM
	Coloquadranoside A	41.13	-1.78	49.23 mM
	Philinopside A	35.12	-5.89	47.74 uM
	Philinopside B	58.71	-7.31	4.41 uM
	Philinopside E	42.78	-9.37	135.51 nM
	Pentactaside B	46.00	-11.83	2.12 nM
	Pentactaside C	36.10	-6.09	34.38 uM
	Fuscocineroside C	28.23	-7.45	3.47 uM
	24-Dehydroechinoside A	34.63	-6.85	9.57 uM
	Saponin	32.97	-8.32	802.70 nM
CB1	Frondoside A	16.12	-6.40	20.37 uM
	Intercedenside A	25.95	-9.66	82.83 nM
	Intercedenside B	19.41	-10.61	16.81 nM
	Intercedenside C	20.80	-14.87	12.65 pM
	Arguside B	15.29	-0.87	230.85 mM
	Arguside C	20.43	-7.16	5.61 uM
	Arguside D	25.81	-6.52	16.74 uM
	Arguside E	27.84	-2.49	14.89 mM
	Impatienside A	8.42	-3.79	1.67 mM
	Nobiliside D	19.49	-9.55	99.42 nM
	Scabraside A	19.81	-9.58	94.32 nM

	Scabraside B	23.73	-11.07	7.66 nM
	Scabraside D	23.58	-7.51	3.12 uM
	Coloquadranoside A	23.25	-3.03	5.97 mM
	Philinopside A	15.97	-4.79	307.95 uM
	Philinopside B	10.08	-4.19	855.13 uM
	Philinopside E	19.94	-9.84	61.06 nM
	Pentactaside B	9.81	-11.16	6.60 nM
	Pentactaside C	16.63	-4.07	1.05 mM
	Fuscocineroside C	25.67	-7.56	2.87 uM
	24-Dehydroechinoside A	23.86	+0.54	NA
	Saponin	25.16	-5.42	105.62 uM
CB2	Frondoside A	176.12	-4.38	614.34 uM
	Intercedenside A	166.55	-10.06	41.99 nM
	Intercedenside B	153.95	-11.86	2.03 nM
	Intercedenside C	148.33	-14.04	51.19 pM
	Arguside B	161.29	-1.65	61.49
	Arguside C	160.23	-3.63	2.17 mM
	Arguside D	180.46	-2.96	6.74 mM
	Arguside E	168.69	-4.45	549.13 uM
	Impatienside A	156.35	-2.50	14.71 mM
	Nobiliside D	143.03	-10.69	14.55 nM
	Scabraside A	203.31	-9.11	210.39 nM
	Scabraside B	166.74	-13.43	143.63 pM
	Scabraside D	181.89	-6.02	38.75 uM
	Coloquadranoside A	173.20	-0.54	398.90 mM
	Philinopside A	184.66	-5.33	123.48 uM
	Philinopside B	176.13	-3.78	1.71 mM
	Philinopside E	164.20	-8.82	341.65 nM
	Pentactaside B	171.64	-11.41	4.35 nM
	Pentactaside C	173.45	-9.44	121.16 nM
	Fuscocineroside C	166.11	-3.64	2.16 mM
	24-Dehydroechinoside A	164.37	-0.06	897.83 mM
	Saponin	170.34	-6.92	8.46 uM

TLR4	Frondoside A	53.08	-5.57	82.40 uM
	Intercedenside A	13.78	-10.54	18.78 nM
	Intercedenside B	15.95	-11.80	2.26 nM
	Intercedenside C	25.27	-15.64	3.41 pM
	Arguside B	25.61	+2.84	NA
	Arguside C	46.66	-4.12	951.89 uM
	Arguside D	25.02	-7.84	1.80 uM
	Arguside E	41.01	-3.28	3.93 mM
	Impatienside A	54.65	-3.61	2.27 mM
	Nobiliside D	26.90	-9.82	63.30 nM
	Scabraside A	32.80	-9.80	65.31 nM
	Scabraside B	23.34	-13.86	69.36 pM
	Scabraside D	26.10	-4.99	220.11 uM
	Coloquadranoside A	14.33	-0.85	236.30 mM
	Philinopside A	32.80	-9.80	65.31 nM
	Philinopside B	49.45	-1.87	42.52 mM
	Philinopside E	20.51	-9.11	211.65 nM
	Pentactaside B	23.60	-9.13	202.04 nM
	Pentactaside C	45.91	-1.90	40.60 mM
	Fuscocineroside C	12.44	-5.68	68.87 uM
	24-Dehydroechinoside A	19.15	+0.89	NA
	Saponin	24.20	-5.72	64.25 uM
TLR9	Frondoside A	43.25	-3.73	1.85 mM
	Intercedenside A	51.86	-12.93	332.56 pM
	Intercedenside B	51.93	-11.55	3.43 nM
	Intercedenside C	36.54	-16.52	779.45 fM
	Arguside B	49.23	+1.31	NA
	Arguside C	52.18	-3.88	1.43 mM
	Arguside D	20.93	-3.55	2.52 mM
	Arguside E	40.18	-3.42	3.10 mM
	Impatienside A	40.87	-3.50	2.74 mM
	Nobiliside D	52.02	-9.20	180.95 nM
	Scabraside A	52.85	-5.20	154.96 uM

	Scabraside B	39.65	-13.05	273.01 pM
	Scabraside D	41.60	-6.80	10.40 uM
	Coloquadranoside A	27.03	-0.84	241.64 mM
	Philinopside A	49.99	-4.07	1.03 mM
	Philinopside B	37.88	-2.46	15.71 mM
	Philinopside E	34.40	-9.79	66.83 nM
	Pentactaside B	51.82	-11.61	3.10 nM
	Pentactaside C	36.71	-7.44	3.54 uM
	Fuscocineroside C	29.76	-3.14	4.98 mM
	24-Dehydroechinoside A	24.43	+0.85	NA
	Saponin	15.25	-6.44	19.17 uM
EPCR	Frondoside A	34.33	8.60 mM	8.60 mM
	Intercedenside A	50.32	-12.63	556.78 pM
	Intercedenside B	51.79	-16.64	630.81 fM
	Intercedenside C	42.67	-20.14	1.71 fM
	Arguside B	45.94	-0.92	211.47 mM
	Arguside C	44.40	-4.32	682.06 uM
	Arguside D	42.19	-4.67	380.50 uM
	Arguside E	33.88	-11.42	4.26 nM
	Impatienside A	38.12	-5.08	189.37 uM
	Nobiliside D	46.71	-13.07	264.27 pM
	Scabraside A	49.28	-6.29	24.51 uM
	Scabraside B	42.18	-20.35	1.21 fM
	Scabraside D	38.94	-9.11	211.64 nM
	Coloquadranoside A	45.55	-3.66	2.08 mM
	Philinopside A	47.50	-6.92	8.43 uM
	Philinopside B	40.41	-6.01	39.63 uM
	Philinopside E	46.15	-12.43	778.27 pM
	Pentactaside B	46.84	-18.02	61.62 fM
	Pentactaside C	37.24	-4.77	318.82 uM
	Fuscocineroside C	30.40	-11.77	2.35 nM
	24-Dehydroechinoside A	49.14	+1.42	NA
	Saponin	36.01	-14.98	10.46 pM

mGluR 8	Frondoside A	57.73	+1.28	NA
	Intercedenside A	68.04	-8.64	464.47 nM
	Intercedenside B	60.27	-11.10	7.32 nM
	Intercedenside C	52.62	-13.57	112.39 pM
	Arguside B	37.86	+1.76	NA
	Arguside C	62.98	-2.68	10.82 mM
	Arguside D	27.76	-4.51	497.80 uM
	Arguside E	48.18	-4.04	1.09 mM
	Impatienside A	34.82	-1.37	98.72 mM
	Nobiliside D	77.97	-8.25	892.54 nM
	Scabraside A	47.06	-6.87	9.25 uM
	Scabraside B	41.07	-11.36	4.70 nM
	Scabraside D	83.82	-6.20	28.66 uM
	Coloquadranoside A	42.83	-0.74	286.80 mM
	Philinopside A	40.55	-2.00	34.27 mM
	Philinopside B	52.39	-3.09	5.45 mM
	Philinopside E	30.99	-7.88	1.67 uM
	Pentactaside B	62.53	-8.81	345.69 nM
	Pentactaside C	50.36	-5.26	139.70 uM
	Fuscocineroside C	23.59	-4.60	423.63 uM
	24-Dehydroechinoside A	57.37	+0.73	NA
	Saponin	37.01	-3.86	1.49 mM
PGD2R	Frondoside A	342.716	-1.91	39.72 mM
	Intercedenside A	330.28	-12.47	725.73 pM
	Intercedenside B	285.47	-13.28	185.67 pM
	Intercedenside C	287.32	-13.68	93.62 pM
	Arguside B	300.15	+2.85	NA
	Arguside C	305.11	-1.95	37.16 mM
	Arguside D	302.07	-3.99	1.19 mM
	Arguside E	322.14	-5.51	91.78 uM
	Impatienside A	313.93	-2.67	11.01 mM
	Nobiliside D	286.63	-9.45	117.57 nM
	Scabraside A	340.01	-6.85	9.50 uM

	Scabraside B	292.64	-12.34	901.37 pM
	Scabraside D	290.84	-6.95	8.03 uM
	Coloquadranoside A	327.74	-1.73	54.34 mM
	Philinopside A	290.44	-2.08	29.88 mM
	Philinopside B	304.88	-5.25	142.87 uM
	Philinopside E	328.58	-9.95	50.73 nM
	Pentactaside B	315.24	-8.79	361.03 nM
	Pentactaside C	286.51	-1.93	38.63 mM
	Fuscocineroside C	279.14	-4.05	1.07 mM
	24-Dehydroechinoside A	337.56	+1.65	NA
	Saponin	323.26	-3.54	2.55 mM
TGFBR2	Frondoside A	49.32	-7.08	6.48 uM
	Intercedenside A	40.40	-12.44	764.62 pM
	Intercedenside B	33.48	-12.67	515.06 pM
	Intercedenside C	55.50	-14.16	41.44 pM
	Arguside B	21.17	-2.67	11.09 mM
	Arguside C	50.01	-3.21	4.42 mM
	Arguside D	40.62	-6.57	15.37 uM
	Arguside E	56.38	-5.82	54.39 uM
	Impatienside A	29.80	-3.23	4.30 mM
	Nobiliside D	35.53	-10.14	36.82 nM
	Scabraside A	63.39	-10.28	29.04 nM
	Scabraside B	57.24	-12.48	707.50 pM
	Scabraside D	33.17	-5.08	190.21 uM
	Coloquadranoside A	50.02	-3.14	5.00 mM
	Philinopside A	47.89	-6.05	37.02 uM
	Philinopside B	33.24	-5.75	60.62 uM
	Philinopside E	30.56	-11.12	7.02 nM
	Pentactaside B	59.46	-7.52	3.10 uM
	Pentactaside C	60.51	-9.43	122.49 nM
	Fuscocineroside C	40.67	-4.71	351.43 uM
	24-Dehydroechinoside A	35.90	-0.22	692.75 mM
	Saponin	47.79	-6.95	8.10 uM

mM; miliMolar, uM; microMolar; nM; nanoMolar; pM; picoMolar, fM; femtoMolar
